# Counter-gradient variation and the expensive tissue hypothesis explain parallel brain size reductions at high elevation in cricetid and murid rodents

**DOI:** 10.1038/s41598-023-32498-4

**Published:** 2023-04-06

**Authors:** Aluwani Nengovhela, Catherine M. Ivy, Graham R. Scott, Christiane Denys, Peter J. Taylor

**Affiliations:** 1 Department of Mammalogy, National Museum, Bloemfontein, 9300 South Africa; 2 Department of Zoology, School of Natural and Mathematical Sciences, University of Venda, Thohoyandou, South Africa; 3 Department of Biology, McMaster University, Hamilton, ON L8S 4K1 Canada; 4 Institut de Systématique, Evolution, Biodiversité (ISYEB), Muséum National d’Histoire Naturelle, CNRS, Sorbonne Université, EPHE, Université des Antilles, CP51, 57 Rue Cuvier, 75005 Paris, France; 5 Afromontane Unit, Department of Zoology and Entomology, University of the Free State, Phuthaditjhaba, South Africa

**Keywords:** Evolutionary biology, Evolution

## Abstract

To better understand functional morphological adaptations to high elevation (> 3000 m above sea level) life in both North American and African mountain-associated rodents, we used microCT scanning to acquire 3D images and a 3D morphometric approach to calculate endocranial volumes and skull lengths. This was done on 113 crania of low-elevation and high-elevation populations in species of North American cricetid mice (two *Peromyscus* species, n = 53), and African murid rodents of two tribes, Otomyini (five species, n = 49) and Praomyini (four species, n = 11). We tested two distinct hypotheses for how endocranial volume might vary in high-elevation populations: the expensive tissue hypothesis, which predicts that brain and endocranial volumes will be reduced to lessen the costs of growing and maintaining a large brain; and the brain-swelling hypothesis, which predicts that endocranial volumes will be increased either as a direct phenotypic effect or as an adaptation to accommodate brain swelling and thus minimize pathological symptoms of altitude sickness. After correcting for general allometric variation in cranial size, we found that in both North American *Peromyscus* mice and African laminate-toothed (*Otomys*) rats, highland rodents had smaller endocranial volumes than lower-elevation rodents, consistent with the expensive tissue hypothesis. In the former group, *Peromyscus* mice, crania were obtained not just from wild-caught mice from high and low elevations but also from those bred in common-garden laboratory conditions from parents caught from either high or low elevations. Our results in these mice showed that brain size responses to elevation might have a strong genetic basis, which counters an opposite but weaker environmental effect on brain volume. These results potentially suggest that selection may act to reduce brain volume across small mammals at high elevations but further experiments are needed to assess the generality of this conclusion and the nature of underlying mechanisms.

## Introduction

Brain size variation between and within species has long interested scientists studying cognition or behavioral flexibility in animals^1–4^. Brain size relative to body mass varies at different rates among different mammal groups^5^. Several factors may explain this variability in brain size, including phylogeny, ecology, basal metabolic rate, behavior, sensory complexity, life histories^5–20^ or the interaction of these factors. Larger brains (especially the neocortex) typically carry cognitive advantages^21–23^, and have also been associated with increased habitat complexity, the variety of life styles relating to diet, sociality, and nocturnal activity^5,7,8,24,25^, predation pressure^26^, and life history variables such as longevity, age at maturity and litter size^6,9,27^. However, even though a large brain has been shown to have potential benefits, brain tissue is said to be extremely energetically costly^20,28^, which might constrain brain size in energetically challenging environments.

Rodents are an ideal group to compare brain size variation because they occupy different niches, differing greatly in lifestyles, habitat, diet and activity period. Locomotive pattern can similarly differ among rodents, they can be arboreal, aerial, terrestrial, semiaquatic, burrowing (fossorial) and rock-dwelling^29–32^. Diet is similarly diverse amongst rodents (e.g., herbivorous, omnivorous^33^, insectivorous^34^ and fungivorous^35^). Using 3D reconstructions of virtual endocasts from micro-CT scanning of crania of extant and extinct species, several recent studies have explored allometric, phylogenetic and environmental correlates of brain evolution in the early evolution of rodents and their relatives^36–38^, in squirrel-like rodents^30–32,39^ and in caviomorph rodents^40,41^.

Enlarged brains have been reported in kangaroo rats (Heteromyidae), deer mice (*Peromyscus leucopus*), voles (*Microtus pennsylvanicus*), octodontid rodents (*Tympanoctomys*, *Octodontomys* and *Octomys*), arboreal squirrel-like rodents, the African wading rat (*Colomys gosling*), the mole rat (*Spalax ehrenbergi*)*,* and some southern African squirrels (squirrel-related clade^42^) and mice (mouse-related clade^42^), and these were associated with complex sensory and locomotory functions (e.g. associated with arboreality), urbanization, higher behavioural complexity, habitat and diet types^25,30–32,43–49^. In contrast, smaller brains have also been reported in African molerats (Bathyergidae), gophers (Geomyidae), fossorial squirrel-like rodents and South African porcupines and cane rats (Ctenohystrica^42^) and some mice (mouse-related clade)^25,30,46^. Relative brain size was not found to be associated with social system in African mole-rats^50^, nor in African striped mice (*Rhabdomys*)^51^, although histological differences in the amygdala and hippocampus regions of the neocortex of different mesic and arid species of striped mice (*Rhabdomys*) were found to be associated with sociality rather than spatial memory in lab tests^52^. Nevertheless, although some previous studies have examined brain size in association with various factors, few have distinguished the potential genetic and environmental contributions to brain size variation.

High-altitude environments present numerous challenges that might influence brain size evolution, so high-altitude mammals present an intriguing system for examining genetic and environmental influences on brain size. High altitude is characterized by reduced ambient temperature that increase the metabolic demands of body heat generation in endotherms, but also by reduced availability of oxygen (hypoxia) to support these high demands^53^. These conditions might also be expected to constrain the growth and/or ongoing maintenance costs of neural tissue, which has a high metabolic activity relative to other tissues, consistent with the expensive tissue hypothesis^54^, leading to smaller brain size at high altitude. Conversely, physiological changes at high elevation could also lead to increased brain volume. Hypoxia at high elevation increases brain blood flow and can induce brain swelling, which due to the confines of a fixed endocranial volume can induce pathological increases in intracranial pressure that contribute to altitude sickness in humans and other mammals^55^. Apart from direct environmental effects on the phenotype, it might therefore be advantageous for high-elevation mammals to have larger endocranial volumes to accommodate increases in brain volume and avoid intracranial hypertension. Habitat differences could also influence brain volume, as high-elevation environments can differ in complexity and predation pressure, but such differences may not apply broadly across mountain regions in different parts of the world^56^. Studies in birds suggest that those inhabiting higher altitudes tend to have relatively larger brains^56^ or hippocampal volumes^57^ compared to birds living at lower altitudes, but birds are more tolerant of high-altitude hypoxia than mammals and are not believed to experience increased intracranial pressure or mountain sickness^58^. On the other hand, in accordance with the expensive tissue (brain) hypothesis, Asiatic toads from high altitudes had smaller brains than lower-altitude populations^59^. Environmental harshness linked to colder climates was shown to be positively correlated with brain size in birds^60^ and humans^61,62^. In vertebrates generally, both phenotypic plasticity and genetic adaptation have been shown to be important in physiological and morphological changes associated with high altitude ^53,63,64^. To the best of our knowledge, this study is the first systematic examination of brain size evolution in divergent rodent clades from high-elevation. Within one of these clades (*Peromyscus* species), by comparing both free-living and laboratory progeny of high (4350 m) and low (430 m) populations, we were able to largely separate the effects of high elevation due to genetic adaptation (natural selection, or “nature”) and environmentally-induced phenotypic plasticity (or “nurture”).

The main aim of our study is to describe the variation in endocranial volume (a proxy for brain size^65,66^) in North American cricetid mice (two *Peromyscus* species) and in African murid rodents of two tribes, Otomyini (five species) and Praomyini (four species), with members of both families occupying both relatively high, alpine (> c.3000 m maximum) and lower, non-alpine (< c.2500 m maximum) elevations, to test how elevation affects brain size in mammals. In this study, we defined high-elevation habitats in Africa based on the standard Vegetation Map of Africa^67^, comprising Afroalpine moorlands, shrublands and grasslands which typically occur above the forest line in Africa at c. 2500–3000 m. In North American Peromyscus included in this study, populations were sampled from very discrete elevations at 4350 m (high) and 430 m (low). We tested two distinct hypotheses for how endocranial volume might vary in high-elevation populations: the expensive tissue hypothesis, which predicts that endocranial volumes will be reduced in high-altitude mammals; and the brain-swelling hypothesis, which predicts that endocranial volumes will be increased in high-altitude mammals. Following from this broader comparison of free-living populations of three diverse rodent clades, in one of them, we then used a common-garden experiment with highland (4350 m) and lowland (430 m) *Peromyscus* mice derived from both free-living and lab-bred populations to further examine whether genetic (“nature”) and/or environmental (“nurture”) factors contribute to brain size variation within this particular clade. We acknowledge that our experimental design cannot account for all potential mechanisms involved in brain development and growth, such as environment-genetic and environment-ontogenetic interactions, the latter which can cause brain size reduction in lab-bred mice raised with poor nutrition^68^. However, our lab-bred mice were raised on a common diet and at a common environment (sea level) to reduce such environmental interactions.

## Results

**Figure 1:**
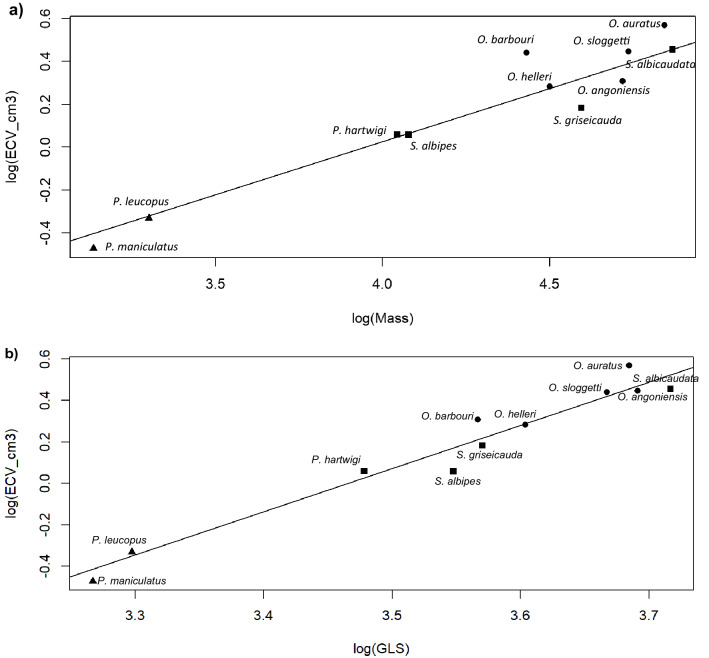
Phylogenetically-controlled regressions (models shown as solid lines) between logged mean species values of endocranial volume (ECV_cm3) and a) body mass and b) skull length (GLS) between three rodent clades; *Peromyscus* (triangles), Otomyini (dots), Praomyini (squares). Means for ECV and skull length were computed from 113 individual crania (Table S1). Mean body masses obtained from literature^71,72^.

Comparing mean values for 11 rodent species, we found significant (*p* < 0.01) correlations between body mass and endocranial volume (ECV) (r = 0.93, df = 9) and between skull length and EVC (r = 0.97, df = 9). With phylogenetic correction (using the tree in Supplementary Fig. S1), we obtained the following regression equations (Fig. 1a,b):

1. Log (ECV) =  − 1.952 + 0.494 [95% CI : 1.71–2.46] × log (mass)
2. Log (ECV) =  − 7.219 + 2.083 [95% CI: 0.36–0.63] × log (skull length)

Most species mean values fell close to the regression lines based on the log-transformed plots (Fig. 1a,b), with the lowest negative residuals associated with *Stenocephalemys* species and the highest positive residuals associated with *O. auratus* and *O. barbouri* (Fig. 1, Fig. S2). Due to the slightly better fit for skull length compared to body mass, as also shown elsewhere for rodents^69^, we used skull length as a proxy for body size rather than body mass in subsequent analyses. No significant differences in residuals (from skull length: Fig. 1b, Supplementary Fig. S2) were obtained between alpine and non-alpine species (t_10_ = 0.306, *p* > 0.05). Since skull length scales to the cube of body mass, the negative (< 1) allometric exponent obtained from our analysis (slope = 2.083/3 = 0.694) falls between the exponent values for endocranial volume scaled to body size across mammals generally (slope = 0.75^10^) and previous data collected across rodents (slope = 0.64^69,70^). When using body mass (equation (2) above), the allometric exponent is 0.494 which seems to underestimate the value expected for rodents^69^.

**Figure 2:**
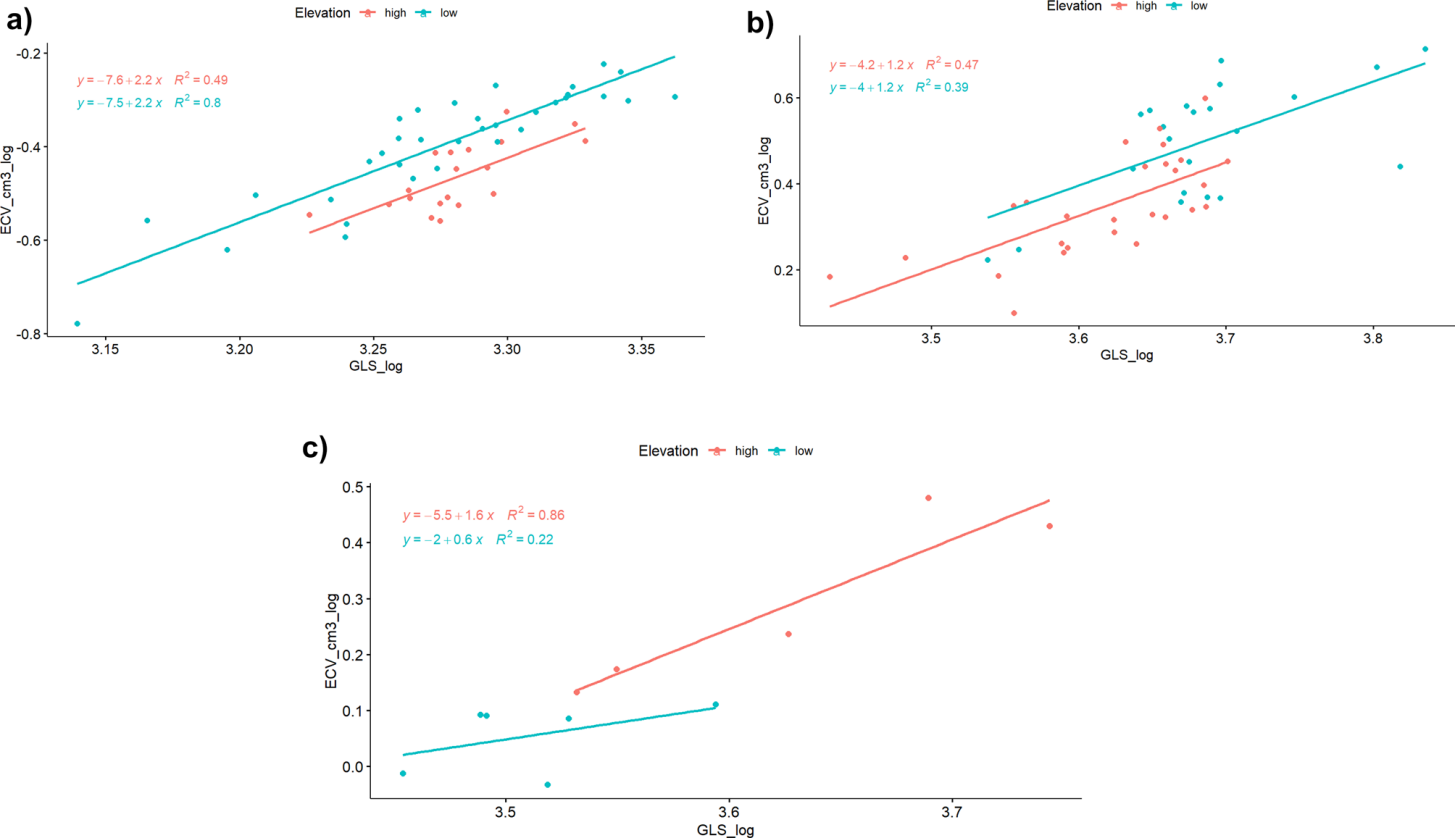
Analysis of co-variance showing regression slopes of log endocranial volume versus log skull length for highland (red) and lowland (blue) (**a**) *Peromyscus* mice (Cricetidae), (**b**) Muridae (Otomyini), (**c**) Muridae (Praomyini). Since length is proportional to the cube of mass (body size), the allometric exponents for body size the slopes in the above regression coefficients are equivalent to one third of the regression coefficients shown. Thus, the allometric exponents for *Peromyscus* (0.7 for high and low populations) are higher than those for *Otomys* (0.4 for high and low species), and Praomyini have different slopes for high (0.5) and low (0.2) species.

After accounting for variation in skull length as a proxy for body size, analysis of covariance showed a significantly smaller adjusted endocranial volume from high-elevation taxa compared with low-elevation taxa for both *Peromyscus* (F_1,51_ = 26.75, *p* < 0.001; slopes homogeneous, F = 0.0007, *p* > 0.05, Fig. 2a) and *Otomys* (F_1,46_ = 4.982, *p* = 0.03; slopes homogeneous, F_1,9_ = 0.007, *p* > 0.05, Fig. 2b). However, some overlap between the low and high elevation individuals of *Otomys* is visible. Although their slopes were homogeneous, (F_1,9_ = 2.124, *p* > 0.05), no statistical differences were observed between the adjusted means of endocranial volume in the Praomyini (F_1,9_ = 2.067, *p* > 0.05) due possibly to the smaller sample size (Fig. 2c).

**Figure 3:**
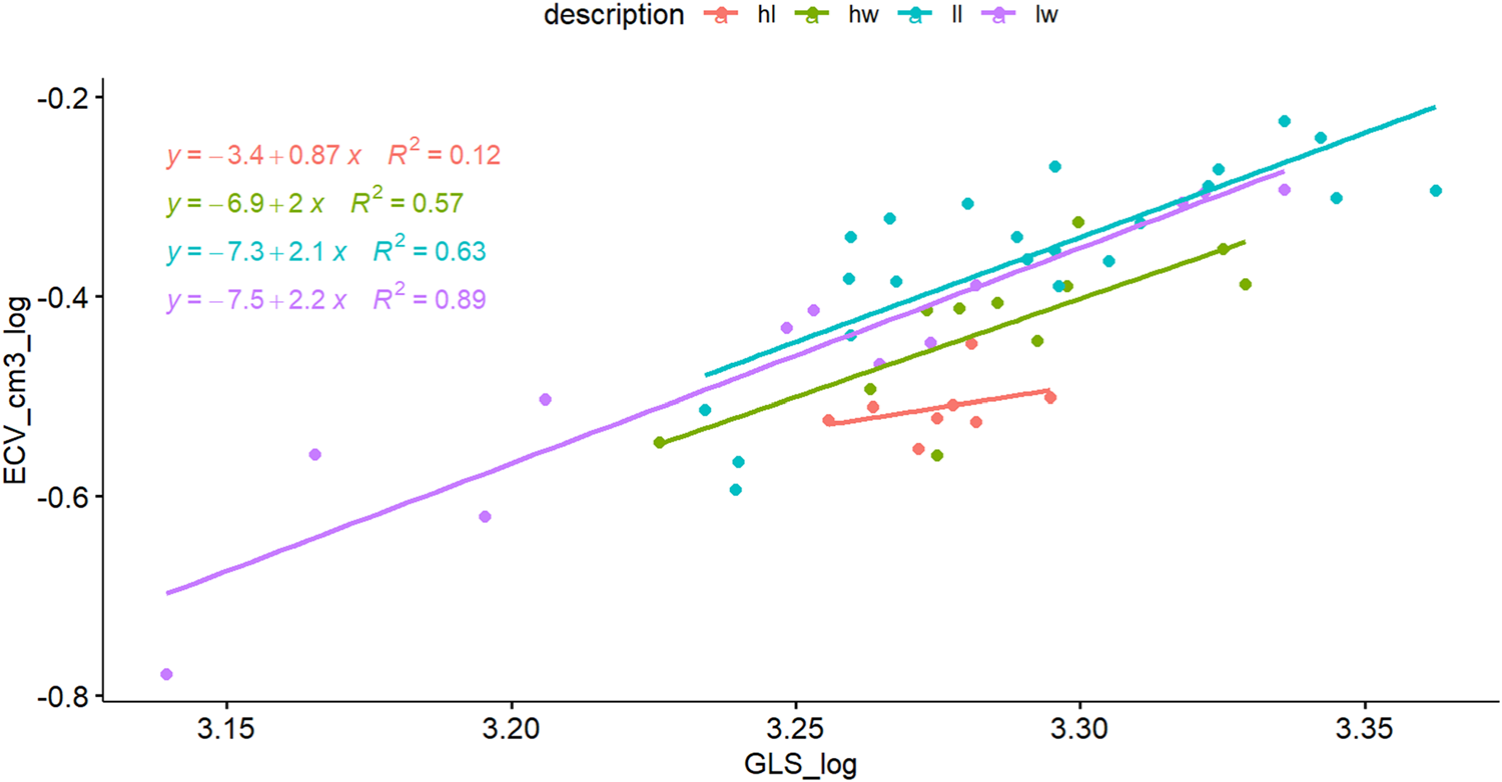
Analysis of co-variance showing regression slopes of log endocranial volume versus log skull length for high-elevation wild-caught (green) and lab-bred (red) and low-elevation wild-caught (purple) and lab-bred (blue) *Peromyscus*.

In the case of *Peromyscus*, crania were obtained both from wild-caught mice from high and low elevations as well as those bred in common-garden lab conditions at sea level from parents caught from either high or low elevations. Crania of these four groups differed significantly in endocranial volume (F_3,48_ = 15.66, *p* < 0.001, Fig. 3), while the slopes of the relationship to skull length were homogeneous (F = 2.065, *p* > 0.05). The largest and smallest adjusted means were for lab-born mice from low elevation and high elevation, respectively. Intermediate values were obtained for wild-caught mice from high and low elevations, with some overlap observed between these groups, but those from low elevation still had higher endocranial volumes than those from high elevation (Fig. 3). Posthoc pair-wise tests (using the Benjamini–Hochberg FDR test with the emmeans test function in the R package “rstatix”) between adjusted means show significant differences between all pairs of groups except for lowland laboratory and wild groups where some overlap was observed (Fig. 3).

## Discussion

### Expensive-tissue hypothesis (ETH)

Although large brains confer cognitive benefits^73^, the large amount of energy required for developing and maintaining brain tissue^74^ should impose serious constraints on brain size evolution^54,75,76^. The ‘expensive-tissue hypothesis’ has been used to interpret trade-offs in which an increase in the size of a metabolically expensive tissue is offset by a decrease in the size of other metabolically expensive tissues^28,77^. For example, a negative relationship has been observed between brain size and testes size (both metabolically expensive organs) in bats^78^ although this was not found to apply to mammals generally^54^. Similarly, no negative correlation was found between brain size and digestive tract length in small mammals^79^. The expensive-tissue hypothesis could apply to any situation where resource limitations constrain the growth and/or ongoing maintenance costs of a tissue and could explain situations in which the advantage of having a larger brain is offset by the disadvantage of needing to supply the resources (O_2_ and metabolic fuels) for growing and supporting the ongoing maintenance costs of a larger brain. Our results showing smaller brains in both *Peromyscus* and *Otomys* at high (alpine) elevations compared to lower-elevation populations are consistent with this explanation. Supporting this, previous studies in *Peromyscus* mice have shown that the masses and physiological capacities of organs involved in partitioning O_2_ and metabolic fuels are larger in populations from high elevation compared to their low-elevation counterparts^80–83^, suggesting that such a trade-off may arise in challenging high-altitude environments.

### Brain-swelling hypothesis

At high elevation, organisms are commonly confronted with disorders such as brain swelling and acute mountain sickness/altitude sickness^84^. Moreover, these disorders bring about learning and memory deficits in organisms and compromise their performance of highly demanding mental functions^85^. For example, when exposed to hypoxic environments, rats exhibited a deficit in spatial working memory^86^. Our data provide some modest suggestion that environmentally-induced plasticity increases endocranial volume in high-elevation deer mice, possibly due to the high-altitude hypoxia effect on brain swelling, as reflected by the difference between wild and lab-raised mice of high-elevation ancestry. Such an effect may only become evident in later development, based on previous reports that mouse embryos from pregnant mothers have reduced brain size when raised in a hypoxic environment^87^. Since endocast volume may not always be directly equivalent to brain volume estimates based on soft tissues, our data do not allow a conclusive test of the brain swelling hypothesis, and further experiments are required to test the applicability of the brain swelling response to hypoxia in high-elevation rodents.

### Effect of genetic origin and environmentally-induced plasticity on highland adaptation of brain size in deer-mice: counter gradient variation?

The pronounced significant difference in endocranial volume between high- and low-elevation populations among laboratory-raised *Peromyscus* mice suggests that there is a strong genetic component to reductions in brain size in high-elevation deer mice as predicted by the expensive tissue hypothesis. However, the attenuation of this difference in mice caught in their native habitats suggests that evolved changes in brain size at high altitude act in opposition to the direct environmental effects on this trait. Such a situation in which selection acts in opposition to environmentally-induced plasticity in a trait is known as counter-gradient variation^63,88–90^. In the case of highland *Peromyscus* mice, the phenotypic effect of hypoxia-induced brain swelling is countered and outweighed by the adaptive genetic effect of decreased brain size predicted by the expensive tissue hypothesis, thereby reducing brain volume to below that observed in lowland mice. As such, the results of our study on brain size conform very closely to a previous theoretical prediction: “For traits that exhibit counteracting environmental and genetic effects, evolved changes in phenotype may be cryptic under field conditions and can only be revealed by rearing representatives of high-and low-altitude populations under standardized environmental conditions to control for plasticity”. The environmental effects on adult brain size in highlanders do not appear to be associated with general variation in growth, because cold hypoxic environments have only slight constraining effects on developmental growth rate that do not affect adult body mass^91^ or its allometric consequences on brain size.

### Life-history correlates of brain size

After correcting for phylogeny and body size, the residual amount of variation in brain size might be explained by species-specific ecology or life history^92^. In general, it is suggested that variation in brain size is associated with habitat type (with forest-dwelling species having relatively larger brains than grassland forms), diet (with omnivores having larger brains than folivores), daily phase of activity (with nocturnal species having larger brains than diurnal), locomotion (arboreal species having larger brains than terrestrial ones), and lifespan (with longer-lived taxa having larger brains than short-lived taxa)^25,46,47,93–95^. However, habitat type did not have clear effects on brain size here, because most of our study species occupy grasslands (*O. auratus*,) or moorlands (*O. sloggetti*, *O. barbouri*, *O. helleri*, *S. albocauda, S. griseicauda* and *P. maniculatus*), and the remaining forest (*S. albipes*, *P. hartwigi*, *P. maniculatus* and *P. leucopus*) and savanna (*O. angoniensis*) species showed both positive and negative residuals to the regressions of brain volume on body mass and skull length (Fig. 1). Daily phase of activity was also unable to explain the observed variation in brain size, because all Praomyini and *Peromyscus* species in our study are nocturnal, and most of these species had slightly smaller brains compared to the generally crepuscular Otomyini species. It is likely that the above-mentioned ecological and life history traits have interactive effects on brain size^25^, a possibility that requires more detailed ecology and life history data to examine fully. Since we did not formulate any testable hypotheses for ecological correlates of brain size in our study we are unable to rule out the importance of these factors which should be considered in future studies.

## Concluding remarks

Convergence between very divergent cricetid and murid rodent lineages from different continents suggests that reductions in brain size at high elevation might be universal among rodents at least but this requires further testing from wider taxonomic and geographic sampling. Our study also highlights the importance of combining the investigation of brain size in wild populations with common-garden experiments for answering questions of “nature” (genetics) and “nurture” (environment) in brain evolution. This study provides the first example of counter-gradient variation in a mammal, whereby the much smaller brain size of lab progeny of highland compared to lowland *Peromyscus* mice (presumably due to natural selection acting to save metabolic costs of maintaining large brains in hypoxic environments) is attenuated in nature by an environmental effect acting in an opposite direction, possible due to brain-swelling induced by hypoxia. Both the expensive tissue and brain swelling hypotheses involve complex interactions between the phenotype, genotype, environment and ontogeny, not all of which could be considered in this study. It could be argued that the improved diet under laboratory conditions compared to wild populations could have biased our results. However, we would counter this argument, because, firstly, laboratory populations are both smaller sized (high) and larger sized (low) than wild populations, and secondly, under constant laboratory conditions, we find the most extreme (genetic) differences in brain size. Nevertheless, we believe that further experiments with stricter controls and more detailed physiological and morphological measurements (including soft tissues of the brain) could provide further insights into the role of environmental, genetic and ontogenetic interactions underlying brain size evolution in rodents and mammals generally. Further insights could also be provided by analysing brain size in populations of mice acclimated to high-altitude hypoxic or cold climates. Such an approach has already been conducted for physiological studies^63^ and could easily be extended in future to include analysis of cranial images from such studies.

## Methods

### Study samples

A total of 113 crania were extracted from 11 rodent species from the Cricetidae from Nebraska and Colorado in the USA (two species of *Peromyscus*), and two African murid Tribes Praomyini (four species, three of *Stenocephalemys* and one of *Praomys*) and Otomyini (five species of *Otomys*) (Table 1, Table S1). For species within the Cricetidae and Tribe Otomyini, sample sizes varied from n = 7 to 27 but species sample sizes were smaller in the Tribe Praomyini (n = 2 to 3). The small sample sizes for Praomyini species was due to the poor availability of specimens from museum collections at the time of study. Muridae specimens were loaned from the Ditsong National Museum of Natural History (DNMNH; Pretoria) and Museum National d’Histoire Naturelle (MNHN; Paris), while the Cricetidae were new collections from the field by McMaster University in Canada. Skull length (GLS) was measured with Avizo by placing a straight-line distance (ruler) from the anterior to the posterior part of the skull (see Supplementary Table S1 for raw data).

**Table 1:** Details of samples used in the study.

Species	Altitude (category)	Country (locality)	Altitude range (m asl)	Collection	N (M,F,?)
*Peromyscus maniculatus*	High	USA (Mount Evans, Clear Creek County, Colorado)	4350	McMaster	9,10,0
*Peromyscus maniculatus*	Low	USA (Nine Mile Prairie, Nebraska)	430	McMaster	5,2,1
*Peromyscus leucopus*	Low	USA (Nine Mile Prairie, Nebraska)	430	McMaster	19,4,3
*Praomys hartwigi*	Low	Cameroon (Mount Oku)	c.2500	MNHN	2, 1, 0
*Stenocephalemys albipes*	Low	Ethiopia	c.2000	MNHN	1, 2, 0
*Stenocephalemys albocaudata*	High	Ethiopia (Bale Mts)	3170	MNHN	1, 1, 0
*Stenocephalemys griseicauda*	High	Ethiopia (Bale Mts)	3170	MNHN	1, 2, 0
*Otomys auratus*	Low	South Africa	1000–2100	DNMNH	5, 6, 0
*Otomys sloggetti*	High	South Africa, Lesotho	1600–3000	DNMNH	6, 5, 0
*Otomys helleri*	High	Ethiopia (Bale Mts)	3326	MHNH	4, 5, 0
*Otomys barbouri*	High	Uganda (Mt Elgon)	4132	MNHN	5, 2, 0
*Otomys angoniensis*	Low	South Africa, Zimbabwe, Eswatini, Zambia	600–1500	DNMNH	6, 5, 0

The question mark (?) represent individuals of unknown sex, meters above sea level (m asl).

In the case of Cricetidae, 53 crania were extracted and analyzed. These skulls were collected from animals used in earlier publications on other topics^96,97^. Since some of the specimens did not have gender data, and the frequency of males and females were close to parity, we combined both sexes in the final analyses to increase our sample size. Adult mice (*Peromyscus*) were live trapped at high altitude (deer mice, *P. maniculatus*) on the summit of Mount Evans (Clear Creek County, CO at 39°35′18’’N, 105°38′38’’W, 4,350 m above sea level) and at low altitude (deer mice, *P. maniculatus*, and white-footed mice, *P. leucopus*) in the Great Plains (Nine Mile Prairie, Lancaster County, NE at 40°52′12’’N, 96°48′20.3’’W, 430 m above sea level) as previously described^64^. Mice were trapped using the Sherman live traps. One set of mice from each locality was euthanized, and crania extracted, within 1–2 days of capture at their native elevation. Another set of mice was transported to a common-garden lab environment at McMaster University (~ 50 m above sea level) and used as parental stock to establish captive bred lines of highland deer mice, lowland deer mice, and lowland white-footed mice. From each of these lines, we euthanized and extracted crania from first-generation progeny from several families, all of which were born and raised to adulthood in a common lab environment. Lab-raised mice were held in standard holding conditions at 25 °C with ambient sea level O_2_ levels, and were provided with unlimited access to mouse chow and water (12 h light:12 h dark photoperiod). For sampling, mice were euthanized by isoflurane overdose followed by decapitation, skulls were prepared using cold water maceration. The epidermal layer was manually removed, and the skulls were submerged in water for three days to soften muscle tissue. Muscle was removed using forceps and the brain was removed using a wide-bore syringe. Skulls were then left to dry. Animal husbandry and experimentation for *Peromyscus* mice followed the ARRIVE guidelines and the guidelines established by the Canadian Council on Animal Care and were approved by the McMaster University Animal Research Ethics Board (ethics number AUP#16–01-02). We confirm that all methods on *Peromyscus* mice were carried out in accordance with relevant guidelines and regulations.

In the case of 60 crania of African Muridae (Otomyini, n = 49 and Praomyini, n = 11) loaned from the DNMNH and MNHN mammal collections, individuals of different species were allocated to “low” and “high” altitudinal categories, generally coinciding with montane/sub-montane (< 2500 m maximum) and alpine/subalpine (> 3000 m maximum) zones respectively, based on literature^98^ and the localities of sampled specimens (Table 1). Since previous studies did not find evidence of sexual dimorphism^99^, we combined the sexes to increase sample sizes. Within the Praomyini, individuals belonging to two species (*Stenocephalemys albocauda* and *S. griseicauda*) were categorized as “high altitude”, from alpine moorland habitats > 3000 m in the Bale Mts of Ethiopia, while two species classified as “low altitude”, from montane forests from mid-altitudes around 2000–2500 m above sea level in Ethiopia (*S. albipes*) and Mt Oku in Cameroon (*Praomys hartwigi*)^98^. Within the Otomyini, individuals of two species were classified as “low altitude”, *Otomys angoniensis* from savanna and grassland habitats in mountain foothills in Southern and East Africa (600–1500 m) and *O. auratus* from grassland habitats on mid-slopes of the Drakensberg Mountains and Eastern Highlands of Zimbabwe in Southern Africa (100–2100 m), while individuals from three alpine moorland species were classified as “high altitude”, *O. helleri* from the Bale Mts of Ethiopia (3300 m), *O. barbouri* from Mt Elgon in Uganda (4100 m) and *O. sloggetti*, mostly restricted to alpine moorlands at 2500–3000 m in the South African and Lesotho Drakensberg-Maloti Mountains (Table 1)^98^.

### Micro CT 3D scanning protocol and endocast reconstruction

Specimens housed at the DNMNH, those collected by McMaster University and some from MNHN were scanned at the South African Nuclear Energy Corporation (NECSA) tomography center, using the X-Tek (Metris) H225L industrial micro-XCT scanner. The remaining specimens from MNHN, were scanned with the Phoenix Nanotom 180 scanner from the FERMAT Federation at the Centre Inter-Universitaire de Recherche et d’Ingenierie des Materiaux (CIRIMAT) in the University of Toulouse Paul Sabatier. Their isometric voxel size ranged from 19–26 µm. Scanner settings of 100–130 kV and 100-180µA were used^100^ (see Supplementary Table S2 for more details).

For endocranium extraction and reconstruction, a specifically developed software for automatic virtual extraction of endocast "Endex" software^101^ (http://liris.cnrs.fr/gilles.gesquiere/wiki/doku.php?id¼endex) was used. Before importing data to Endex, the surface of the entire skull was generated without smoothing and saved in “obj” format in Avizo v8.0 (Supplementary Fig. S3; lateral images of crania shown in top left panel for examples of each genus). This was imported into Endex and converted to "point cloud". The point cloud was then opened in Endex and a sphere was inserted and positioned at the centre of the intracranial space. The sphere then underwent several phases of expansion until it approached as close as possible the internal surface of the bone. Once the deformation of the sphere was completed, its mesh was exported in “obj” format and then converted to “ply” format by MeshLab v1.3.2. The final step involved the refinement of the automatic segmentation by converting the surface of the endocrania into 2D slices under Avizo v8.0. with the use of "Scan Surface to Volume" module. These sections were then merged with the segmented bone sections with the module "Arithmetic", which allowed the correction of zones where the automatic segmentation has crossed closed regions (where bone is possibly thin). Because Endex was generated for hominin endocast reconstruction, its use on rodents was limited. The Endex sphere could not access some parts of the rodents’ endocranium. Therefore, the slices that were not accessed by the Endex sphere but still part of the endocranium were manually segmented in Avizo. In each slice, the endocranial cavity was coloured and attributed to the material representing the endocranium (Supplementary Fig. S3; endocranial cavities shown in red within surrounding cranial tissue in lateral views for examples of each genus). Dorsal, ventral and lateral views of the complete, extracted 3D endocrania are shown in Supplementary Fig. S3.

### Phylogenetic sampling and analysis

Cytochrome b sequences used were downloaded from GenBank and one sequence per species was used. The sequences were first aligned with CLUSTALW and a phylogram tree based on the maximum composite likelihood method^102^ was generated using MEGA7^103^ software, with an assumption that all the species have been identified correctly^104^. A neighbour-joining phylogenetic tree^105^ was generated (Fig. S1). The tree was used for phylogenetic correction among our samples (see Fig. 1) using Phylogenetic Generalized Least Squares (PGLS) in R studio.

### Estimation of endocranial volume (ECV), body mass and skull length

Measurements of ECV were computed from the unsmoothed 2D segmentation labels using the “Material statistics” module available on Avizo v8.0 (see Supplementary Fig. S3 for different views of the endocasts used to calculate ECV).

The greatest skull length (in mm) was calculated from dorsal views of each 3D cranial image in Avizo v8.0, taken in a straight line from the anterior midpoint of the nasal bone to the posterior-most mid-dorsal surface of the occipital bone. Mean body masses (g) were obtained for each species from the literature^71,72^. The raw data for ECV and skull length are shown in Table S1 (n = 113).

### Statistical analyses

Residuals of ECV from Analysis of Covariance (ANCOVA) models were tested for normality in three samples comprising *Peromyscus* (n = 53), Otomyini (n = 49) and Praomyini (n = 11), by using the Shapiro-Wilks test. In all cases, distributions conformed to normality. We used the R ‘Geiger’ and ‘phytools’ packages for phylogenetic bivariate linear regression (PGLS) and non-phylogenetic least squares regressions (GLS) of mean species values for ECV against both body mass and skull length to account for phylogeny and allometry (R4.2.2, R Development Core Team, 2022). To assess inter-species allometric trends across murid and cricetid rodents in ECV, we conducted PGLS and GLS on mean species values of ECV versus body mass (calculated for species means from the literature) and skull length (calculated from greatest skull lengths obtained for cranial images in Avizo; see above) in the eleven rodent species (n = 113, Table 1, Table S1). For further intraspecific analysis, we used skull length as opposed to body mass since a) interspecific regressions gave stronger correlations with ECV for skull length compared to body mass, and b) previous studies showed that body and/or skull length were better predictors of body size than body mass in mouse-related rodents^69^. To examine the effects of allometry and elevation on ECV at a finer taxonomic scale, in each of the three clades (*Peromyscus*, Otomyini and Praomyini), we used Analysis of Co-variance (ANCOVA) using both PAST^106^ and the R packages ’emmeans’, ‘rstatix’, ‘car’ and ‘ggpubr’, with skull length as covariate, ECV as the response variable and elevation as the group variable. In all cases, the slopes of EVC versus skull length in high versus low elevation populations were homogeneous, allowing meaningful comparisons of adjusted mean ECV in high and low populations of each taxon-sample (see Results). All plots were computed using the R packages “ggplot2” and “ggpubr”. The R-script and data input are contained in the Supplementary Information.

## Supplementary Information

Supplementary Information.

## Data Availability

The raw data for skull lengths and endocranial volumes are provided in Supplementary Table S1. The R-script used in this study, is provided as Supplementary File S6 (R Studio script). The 3D reconstructed image stacks of skulls are uploaded to MorphoSource (https://www.morphosource.org/dashboard/my/media?locale=en).
