# Supplementary material for: Counter-gradient variation and the expensive tissue hypothesis explain parallel brain size reductions at high elevation in cricetid and murid rodents
Source: Sci Rep. 2023 Apr 6;13:5617. doi: 10.1038/s41598-023-32498-4 (PMC10079977; doi:10.1038/s41598-023-32498-4)
Supplement: Supplementary file 1 — Supplementary Information. [file 41598_2023_32498_MOESM1_ESM.docx]

**Counter-gradient variation and the expensive tissue hypothesis explain parallel brain size reductions at high elevation in Cricetid and Murid rodents**

Aluwani Nengovhela*, Catherine M. Ivy, Graham R. Scott, Christiane Denys, Peter J. Taylor

**Supplementary Information**


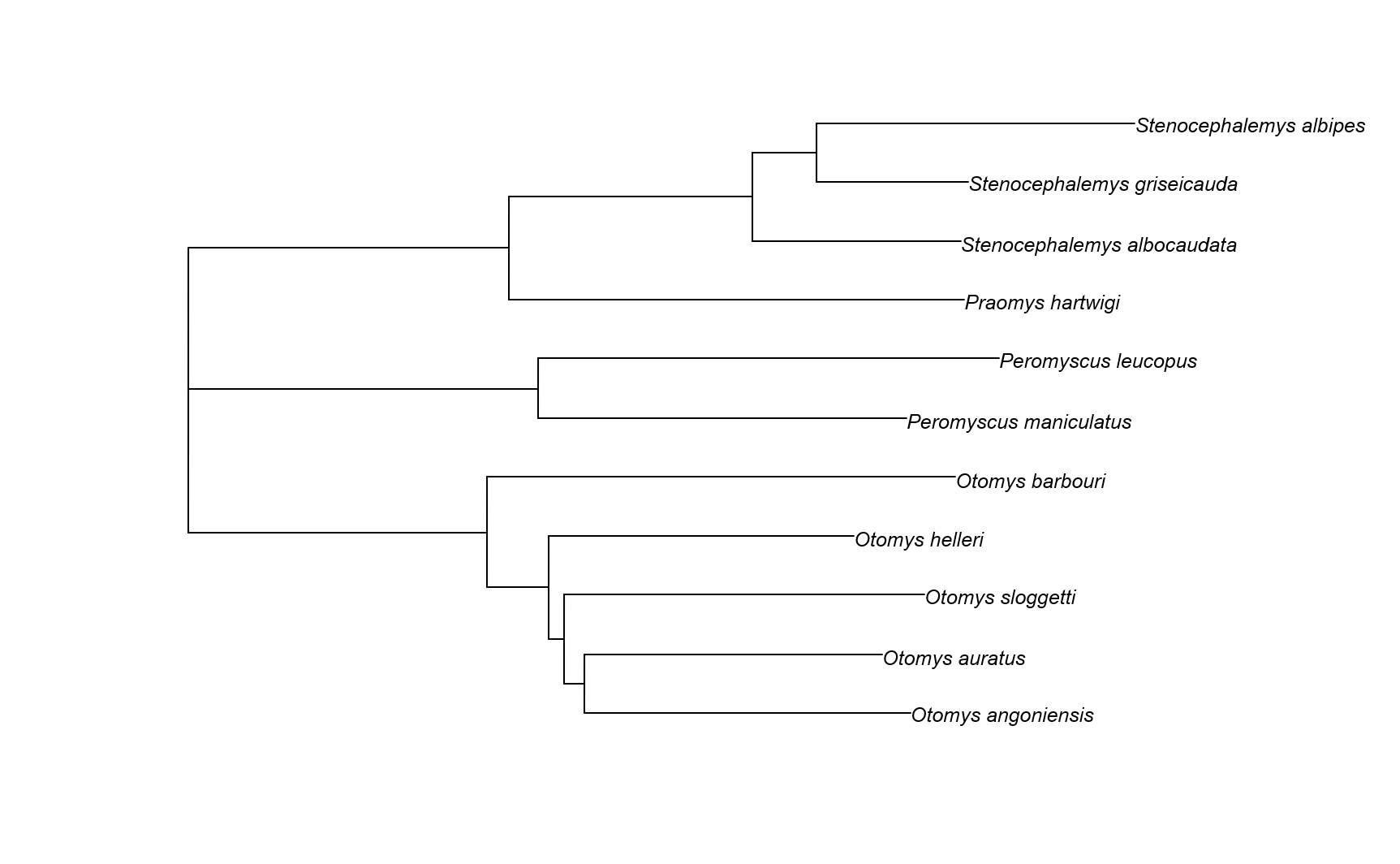


**Figure S1**. Phylogram tree produced by neighbour-joining method with branch length. Cytochrome b sequences obtained from the GenBank.

**Figure S2.** Residuals for 11 rodent species from the phylogenetical regression model of ECV with skull length from Fig. 1b. Full details for species explained in Table 1.


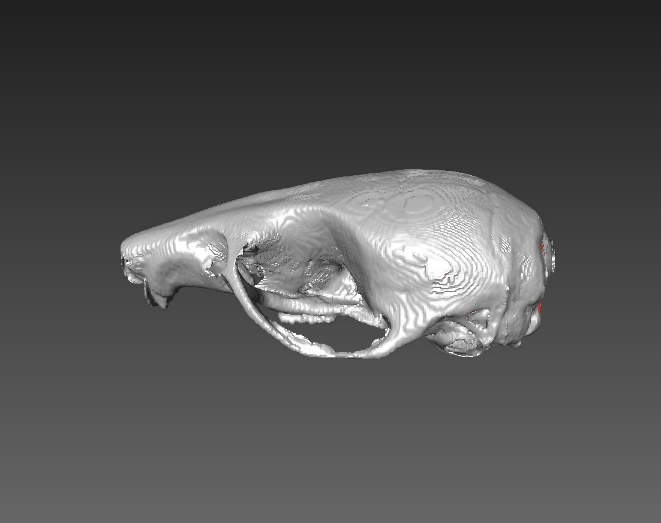

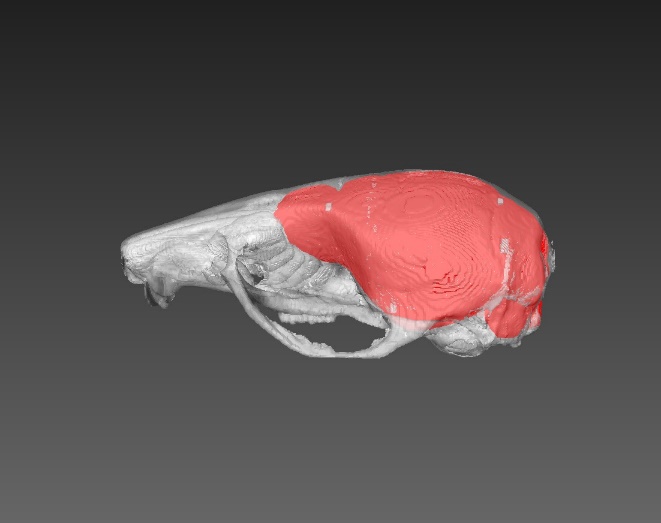

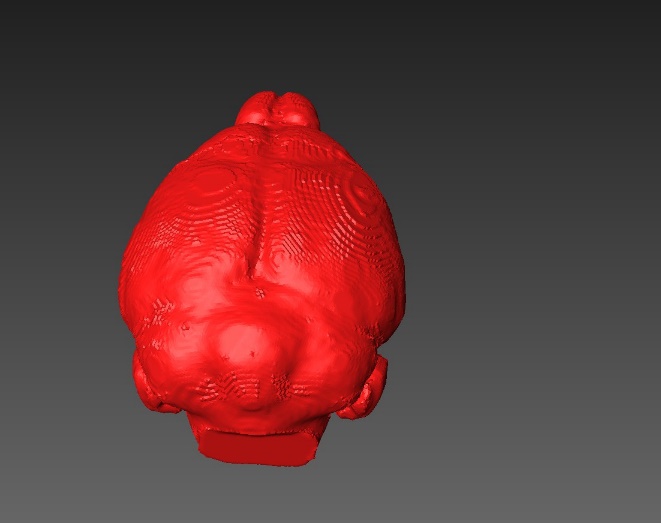


*Peromyscus*

a)


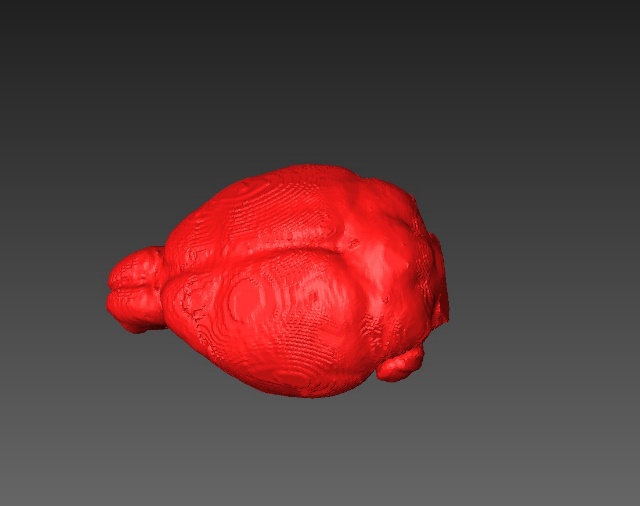

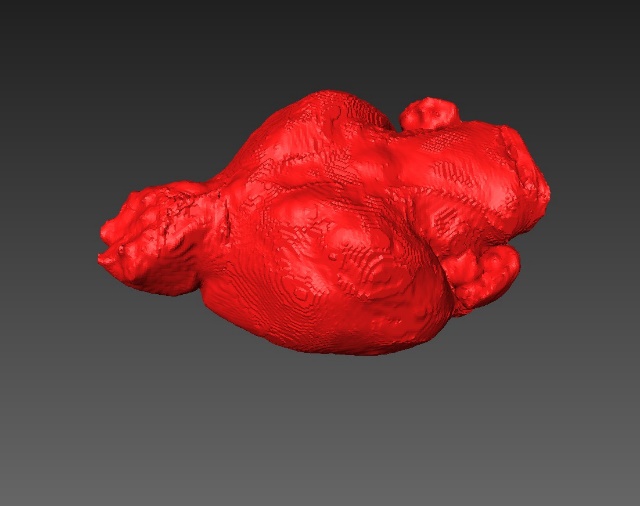

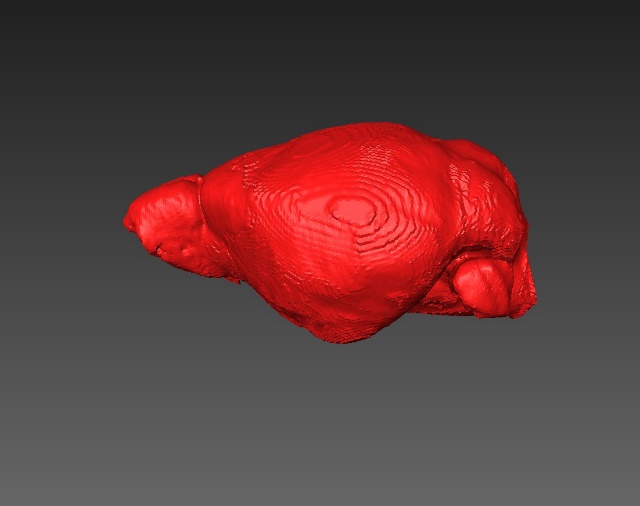


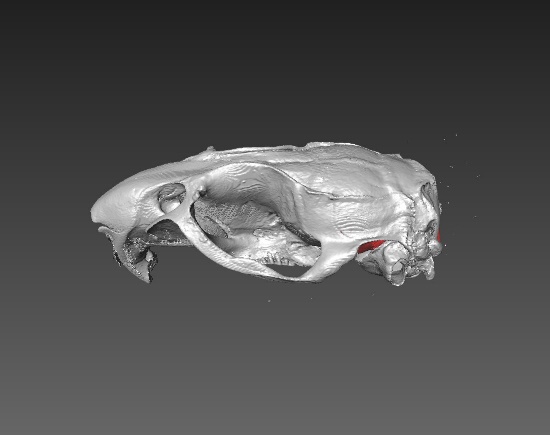

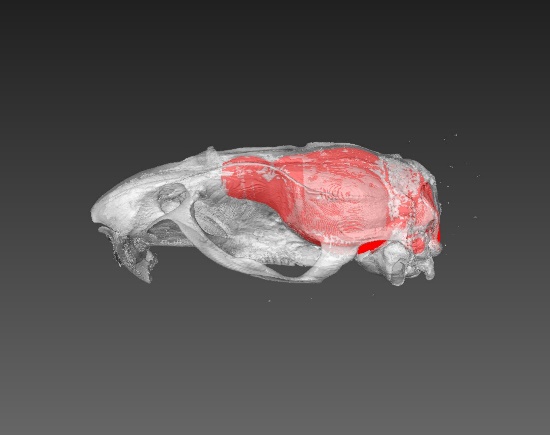

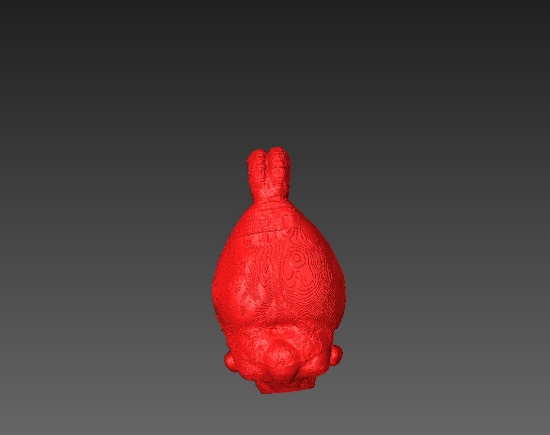


*Otomys*

b)


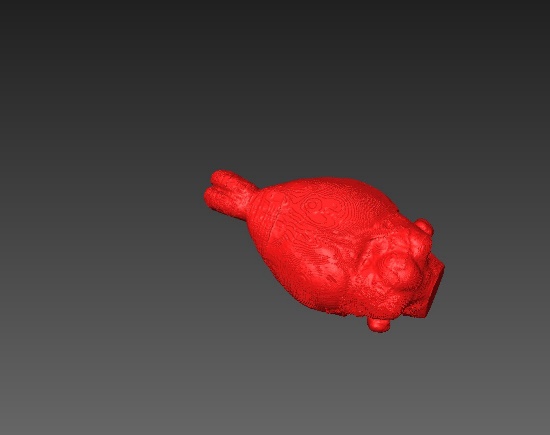

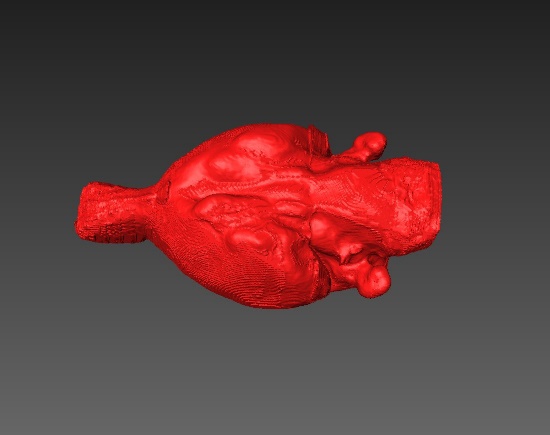

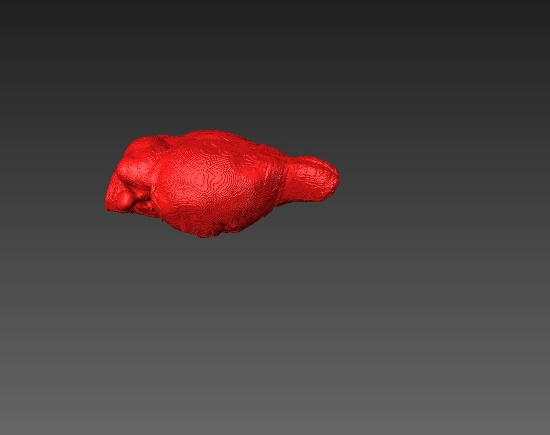


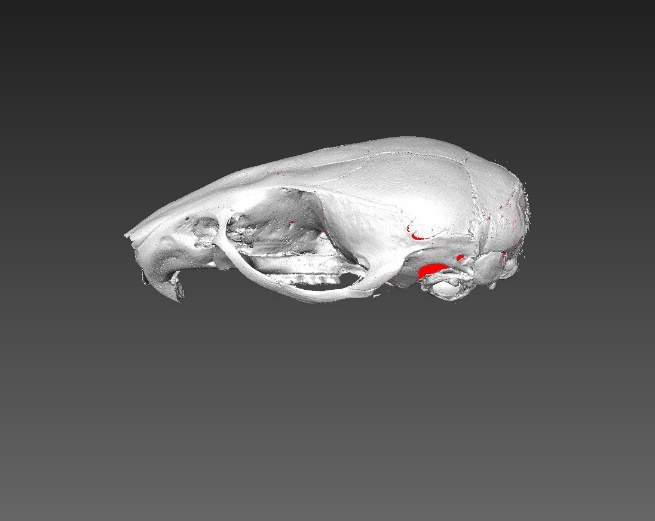

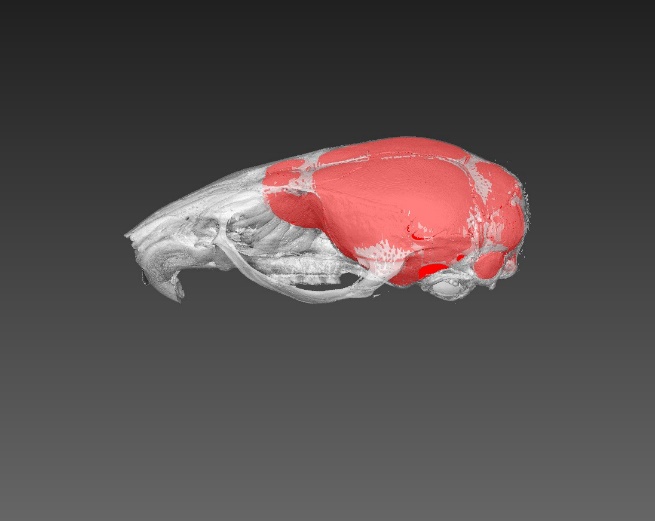

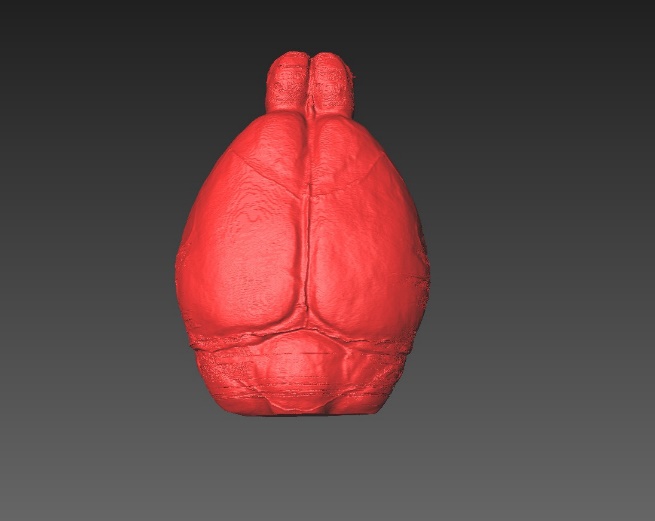


*Praomys*

c)


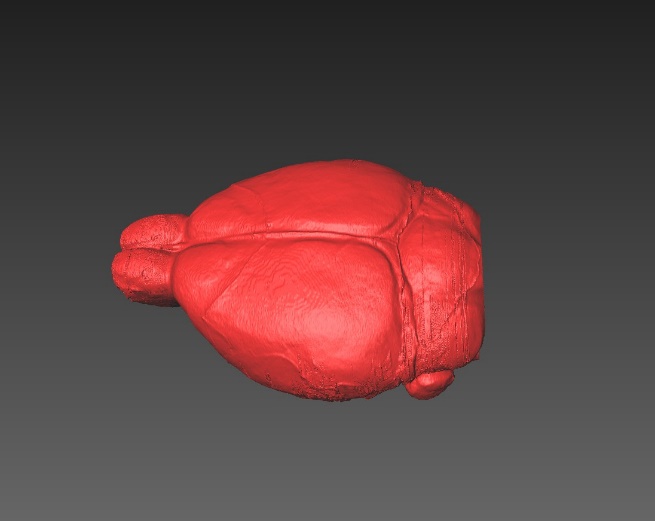

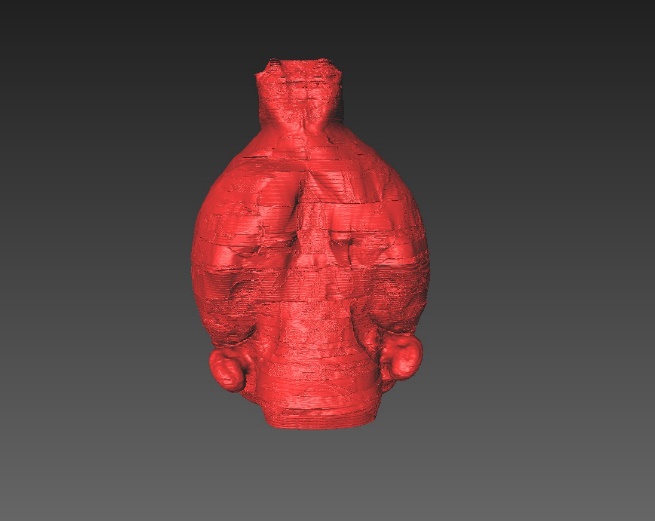

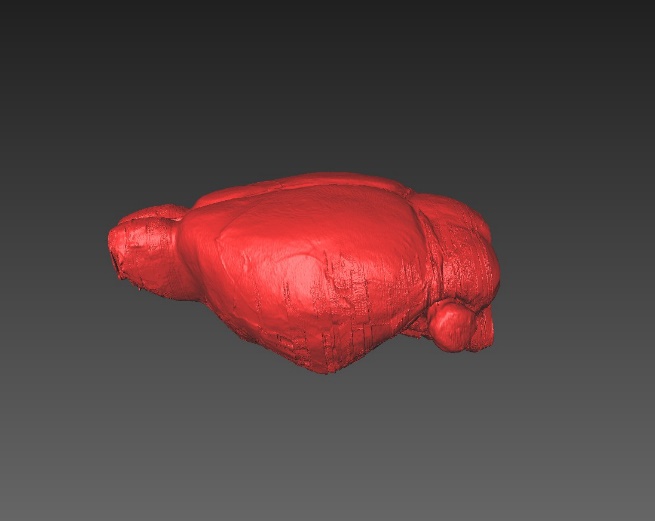


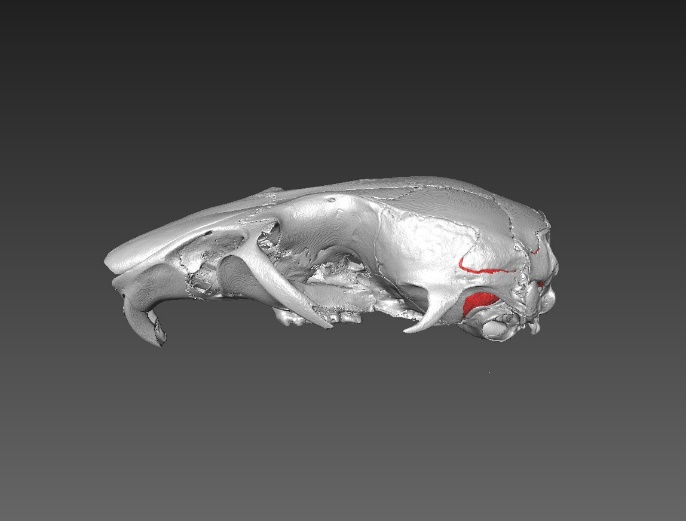

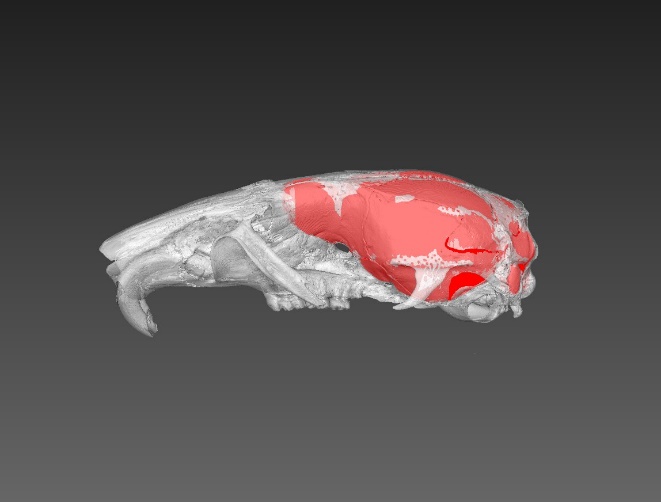

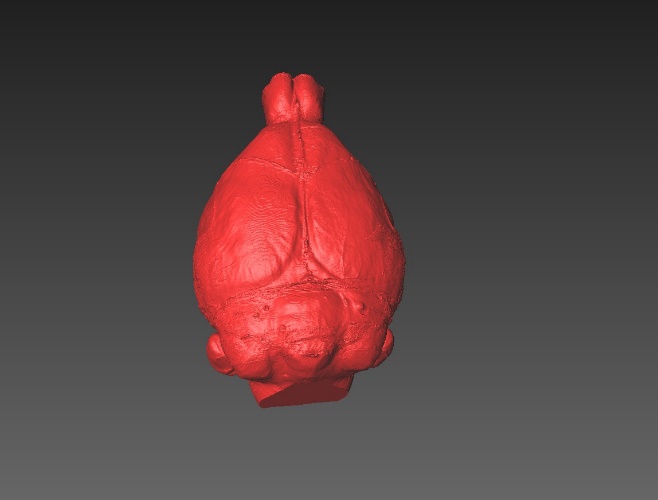


*Stenocephalemys*

d)


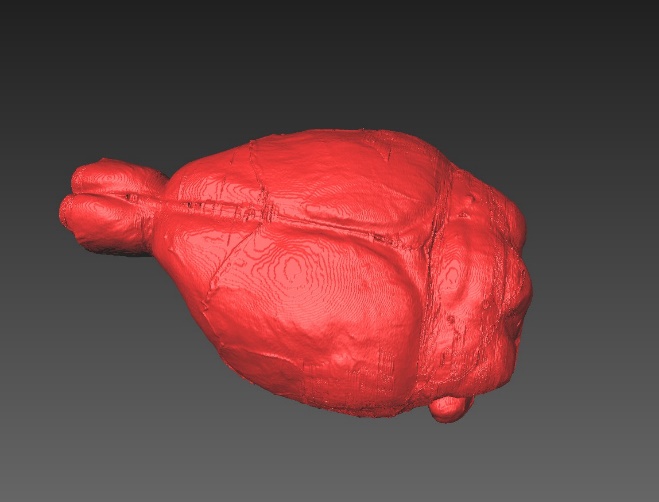

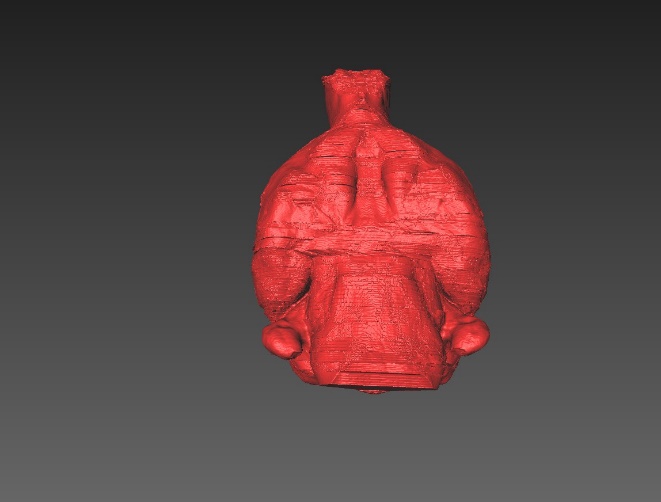

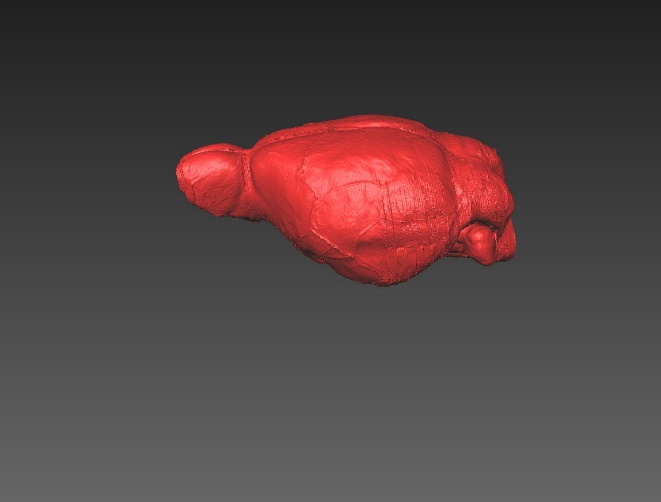


**Figure S3.** Different views of the skulls and endocasts visualized in Aviso v8.0 and used to calculate endocranial volume (ECV) for each genus a) *Peromyscus*, b) *Otomys*, c) *Praomys* and d) *Stenocephalemys*.

Table S1. Raw data used in the study with endocranial volume (ECV), skull length (GLS), elevation and description indicated.

| Species name | Collections number | Sex (g) | Skull length (GLS_mm) | ECV_cm3 | Elevation | Description | Country |
| --- | --- | --- | --- | --- | --- | --- | --- |
| *Otomys angoniensis* | TM103 | Female | 39.22 | 1.430067627 | Low | N/A | South Africa |
| *Otomys angoniensis* | TM123 | Female | 46.3 | 2.042627686 | Low | N/A | Zimbabwe |
| *Otomys angoniensis* | TM22700 | Male | 39.93 | 1.446628418 | Low | N/A | South Africa |
| *Otomys angoniensis* | TM22876 | Male | 37.97 | 1.546006958 | Low | N/A | Swaziland |
| *Otomys angoniensis* | TM24415 | Male | 34.4 | 1.250133398 | Low | N/A | South Africa |
| *Otomys angoniensis* | TM25623 | Male | 40.29 | 1.443271118 | Low | N/A | South Africa |
| *Otomys angoniensis* | TM35027 | Male | 39.3 | 1.460794189 | Low | N/A | Zimbabwe |
| *Otomys angoniensis* | TM44987 | Female | 38.17 | 1.754024048 | Low | N/A | Swaziland |
| *Otomys angoniensis* | TM4504 | Male | 45.52 | 1.55356543 | Low | N/A | South Africa |
| *Otomys angoniensis* | TM45584 | Female | 35.13 | 1.2808 | Low | N/A | South Africa |
| *Otomys angoniensis* | TM6485 | Female | 44.8 | 1.957693115 | Low | N/A | Zambia |
| *Otomys auratus* | TM10282 | Female | 40.01 | 1.777526367 | Low | N/A | South Africa |
| *Otomys auratus* | TM15012 | Female | 38.92 | 1.65731665 | Low | N/A | South Africa |
| *Otomys auratus* | TM19287 | Male | 40.31 | 1.987977783 | Low | N/A | South Africa |
| *Otomys auratus* | TM2916 | Female | 38.76 | 1.704628296 | Low | N/A | South Africa |
| *Otomys auratus* | TM35182 | Male | 42.36 | 1.825832 | Low | N/A | South Africa |
| *Otomys auratus* | TM3611 | Male | 40.74 | 1.687761475 | Low | N/A | South Africa |
| *Otomys auratus* | TM3614 | Female | 39.45 | 1.570219116 | Low | N/A | South Africa |
| *Otomys auratus* | TM41608 | Male | 39.56 | 1.763111 | Low | N/A | South Africa |
| *Otomys auratus* | TM46864 | Female | 40.27 | 1.880129 | Low | N/A | South Africa |
| *Otomys auratus* | TM7597 | Male | 38.4 | 1.770856323 | Low | N/A | South Africa |
| *Otomys auratus* | TM9052 | Female | 39.38 | 1.78917981 | Low | N/A | South Africa |
| *Otomys barbouri* | PM1933-2758 | Female | 35.32 | 1.429014282 | High | N/A | Uganda |
| *Otomys barbouri* | PM1933-2759 | Female | 32.53 | 1.256418213 | High | N/A | Uganda |
| *Otomys barbouri* | PM1933-2760 | Male | 39.84 | 1.487712402 | High | N/A | Uganda |
| *Otomys barbouri* | PM1933-2762 | Male | 38.28 | 1.552892822 | High | N/A | Uganda |
| *Otomys barbouri* | PM1933-2764 | Male | 36.3 | 1.383681519 | High | N/A | Uganda |
| *Otomys barbouri* | PM1933-2765 | Male | 34.65 | 1.203698364 | High | N/A | Uganda |
| *Otomys barbouri* | PM1933-2766 | Male | 30.9 | 1.201383545 | High | N/A | Uganda |
| *Otomys helleri* | PM1972-218 | Male | 38.82 | 1.562871094 | High | N/A | Ethiopia |
| *Otomys helleri* | PM1972-220 | Male | 36.17 | 1.298947876 | High | N/A | Ethiopia |
| *Otomys helleri* | PM1972-221 | Male | 37.49 | 1.33310144 | High | N/A | Ethiopia |
| *Otomys helleri* | PM1972-223 | Female | 36.22 | 1.271706665 | High | N/A | Ethiopia |
| *Otomys helleri* | PM1972-224 | Female | 37.48 | 1.37260376 | High | N/A | Ethiopia |
| *Otomys helleri* | PM1972-225 | Female | 36.32 | 1.285870361 | High | N/A | Ethiopia |
| *Otomys helleri* | PM1972-226 | Female | 38.06 | 1.296744995 | High | N/A | Ethiopia |
| *Otomys helleri* | PM1972-227 | Female | 35.02 | 1.104815796 | High | N/A | Ethiopia |
| *Otomys helleri* | PM1972-228 | Male | 35.01 | 1.417505615 | High | N/A | Ethiopia |
| *Otomys sloggetti* | TM1203 | Male | 39.22 | 1.577509033 | High | N/A | South Africa |
| *Otomys sloggetti* | TM12915 | Female | 38.47 | 1.389318726 | High | N/A | Lesotho |
| *Otomys sloggetti* | TM13730 | Female | 38.75 | 1.635035645 | High | N/A | Lesotho |
| *Otomys sloggetti* | TM16519 | Male | 39.87 | 1.820356323 | High | N/A | Lesotho |
| *Otomys sloggetti* | TM22664 | Male | 39.53 | 1.404328979 | High | N/A | South Africa |
| *Otomys sloggetti* | TM22670 | Female | 37.78 | 1.644431763 | High | N/A | Lesotho |
| *Otomys sloggetti* | TM22676 | Female | 39.9 | 1.414764893 | High | N/A | South Africa |
| *Otomys sloggetti* | TM22684 | Male | 38.81 | 1.381087036 | High | N/A | South Africa |
| *Otomys sloggetti* | TM7606 | Male | 39.07 | 1.538748535 | High | N/A | South Africa |
| *Otomys sloggetti* | TM7780 | Male | 40.49 | 1.571743164 | High | N/A | South Africa |
| *Otomys sloggetti* | TM7781 | Female | 38.67 | 1.697815796 | High | N/A | South Africa |
| *Peromyscus leucopus* | AN002 | Male | 26.62 | 0.6777441 | Low | Wild | USA |
| *Peromyscus leucopus* | AN003 | Male | 27.01 | 0.6772032 | Low | Lab | USA |
| *Peromyscus leucopus* | AN004 | Male | 26.04 | 0.7118267 | Low | Lab | USA |
| *Peromyscus leucopus* | AN005 | Male | 27.78 | 0.7618431 | Low | Lab | USA |
| *Peromyscus leucopus* | AN006 | Male | 26.99 | 0.7017256 | Low | Lab | USA |
| *Peromyscus leucopus* | LN17B | Male | 25.87 | 0.6609509 | Low | Wild | USA |
| *Peromyscus leucopus* | LN17D | Male | 28.1 | 0.7461515 | Low | Wild | USA |
| *Peromyscus leucopus* | LN17G | Male | 26.41 | 0.6397844 | Low | Wild | USA |
| *Peromyscus leucopus* | LN17H | Male | 27.71 | 0.7441191 | Low | Wild | USA |
| *Peromyscus leucopus* | LN17J | Female | 27.6 | 0.7363071 | Low | Wild | USA |
| *Peromyscus leucopus* | LN17N | Female | 25.75 | 0.6494713 | Low | Wild | USA |
| *Peromyscus leucopus* | LNF1104ak | Female | 28.36 | 0.7395663 | Low | Lab | USA |
| *Peromyscus leucopus* | LNF1112ah | Male | 26.25 | 0.6805291 | Low | Lab | USA |
| *Peromyscus leucopus* | LNF1112aj | Male | 26.03 | 0.6824985 | Low | Lab | USA |
| *Peromyscus leucopus* | LNF112ai | Male | 26.04 | 0.6448302 | Low | Lab | USA |
| *Peromyscus leucopus* | LNF1153a3 | Female | 27.73 | 0.7491603 | Low | Lab | USA |
| *Peromyscus leucopus* | LNF1155bq | Male | 28.28 | 0.7860842 | Low | Lab | USA |
| *Peromyscus leucopus* | LNF1159ag | Male | 27.25 | 0.6948113 | Low | Lab | USA |
| *Peromyscus leucopus* | LNF1159au | Male | 26.81 | 0.7112853 | Low | Lab | USA |
| *Peromyscus leucopus* | LNF1159bn | Male | 26.86 | 0.6961703 | Low | Lab | USA |
| *Peromyscus leucopus* | LNF1160al | Male | 26.99 | 0.7634993 | Low | Lab | USA |
| *Peromyscus leucopus* | LNF1160am | Male | 28.1 | 0.7993542 | Low | Lab | USA |
| *Peromyscus leucopus* | LNF1160aq | Male | 26.58 | 0.7359981 | Low | Lab | USA |
| *Peromyscus leucopus* | LNF124 | - | 28.86 | 0.7456226 | Low | Lab | USA |
| *Peromyscus leucopus* | LNF135 | - | 27.4 | 0.7218648 | Low | Lab | USA |
| *Peromyscus leucopus* | LNF145 | - | 26.22 | 0.7249745 | Low | Lab | USA |
| *Peromyscus maniculatus* | AN001 | Male | 26.17 | 0.6263092 | Low | Wild | USA |
| *Peromyscus maniculatus* | AN007 | - | 25.53 | 0.5679921 | Low | Lab | USA |
| *Peromyscus maniculatus* | AN008 | Female | 25.52 | 0.5525744 | Low | Lab | USA |
| *Peromyscus maniculatus* | AN009 | Female | 25.38 | 0.598588 | Low | Lab | USA |
| *Peromyscus maniculatus* | LN17A | Male | 24.42 | 0.5378608 | Low | Wild | USA |
| *Peromyscus maniculatus* | LN17E | Male | 23.7 | 0.5726445 | Low | Wild | USA |
| *Peromyscus maniculatus* | LN17I | Male | 23.09 | 0.459368 | Low | Wild | USA |
| *Peromyscus maniculatus* | LN17L | Male | 24.68 | 0.6044445 | Low | Wild | USA |
| *Peromyscus maniculatus* | ME0817_001 | Male | 26.39 | 0.6613286 | High | Wild | USA |
| *Peromyscus maniculatus* | ME0817_002 | Female | 26.44 | 0.5721016 | High | Wild | USA |
| *Peromyscus maniculatus* | ME0817_004 | Male | 26.54 | 0.6623459 | High | Wild | USA |
| *Peromyscus maniculatus* | ME0817_005 | Male | 25.18 | 0.5793192 | High | Wild | USA |
| *Peromyscus maniculatus* | ME0817_011 | Male | 27.91 | 0.6784481 | High | Wild | USA |
| *Peromyscus maniculatus* | ME0817_018 | Female | 26.72 | 0.6659232 | High | Wild | USA |
| *Peromyscus maniculatus* | ME0817_022 | Female | 27.8 | 0.7032895 | High | Wild | USA |
| *Peromyscus maniculatus* | ME0817_024 | Female | 27.1 | 0.7224584 | High | Wild | USA |
| *Peromyscus maniculatus* | ME0817_026 | Male | 27.05 | 0.6771785 | High | Wild | USA |
| *Peromyscus maniculatus* | ME0817_046 | Male | 26.13 | 0.6107458 | High | Wild | USA |
| *Peromyscus maniculatus* | ME0817_050 | Female | 26.91 | 0.641103 | High | Wild | USA |
| *Peromyscus maniculatus* | MEF1134at | Female | 26.44 | 0.5935914 | High | Lab | USA |
| *Peromyscus maniculatus* | MEF1146av | Female | 26.97 | 0.6059408 | High | Lab | USA |
| *Peromyscus maniculatus* | MEF1146aw | Female | 26.51 | 0.6014285 | High | Lab | USA |
| *Peromyscus maniculatus* | MEF1146ax | Female | 26.35 | 0.5757155 | High | Lab | USA |
| *Peromyscus maniculatus* | MEF1148d | Male | 26.6 | 0.6392412 | High | Lab | USA |
| *Peromyscus maniculatus* | MEF1149aL | Male | 25.94 | 0.5925216 | High | Lab | USA |
| *Peromyscus maniculatus* | MEF1149am | Male | 26.14 | 0.6002584 | High | Lab | USA |
| *Peromyscus maniculatus* | MEF1149L | Female | 26.62 | 0.5916082 | High | Lab | USA |
| *Praomys hartwigi* | MNHN CG 2011_218 | Female | 31.62 | 0.9878971 | Low | N/A | Cameroon |
| *Praomys hartwigi* | MNHN CG 2011_221 | Male | 32.83 | 1.094851 | Low | N/A | Cameroon |
| *Praomys hartwigi* | MNHN CG 2011_236 | Male | 32.74 | 1.096526 | Low | N/A | Cameroon |
| *Stenocephalemys albipes* | MNHN 1972_167 | Male | 36.38 | 1.117667 | Low | N/A | Ethiopia |
| *Stenocephalemys albipes* | MNHN 1972_186 | Female | 34.06 | 1.089158 | Low | N/A | Ethiopia |
| *Stenocephalemys albipes* | MNHN 1972_187 | Female | 33.74 | 0.9682756 | Low | N/A | Ethiopia |
| *Stenocephalemys albocaudata* | MNHN 1972_208 | Male | 40.01 | 1.616297 | High | N/A | Ethiopia |
| *Stenocephalemys albocaudata* | MNHN 1972_217 | Female | 42.24 | 1.536755 | High | N/A | Ethiopia |
| *Stenocephalemys griseicauda* | MNHN 1972_155 | Male | 37.58 | 1.266717 | High | N/A | Ethiopia |
| *Stenocephalemys griseicauda* | MNHN 1972_158 | Female | 34.8 | 1.189752 | High | N/A | Ethiopia |
| *Stenocephalemys griseicauda* | MNHN 1972_162 | Female | 34.18 | 1.142264 | High | N/A | Ethiopia |

Table S2. Settings of the CT scanner as well as the location of the scanner and date of each scan.

| Subfamily/tribe | Segmented taxa | Collection number | Sex | Location of CT scanner and date of the scan | Energy settings  Kv µA | | Number of views | Interslice spacing/interpixel distance (mm) |  |
| --- | --- | --- | --- | --- | --- | --- | --- | --- | --- |
| Otomyini | *Otomys angoniensis* | TM103 | Female | Necsa - 2016 | 110 | 100 | 1001 | 0.022556 |  |
| Otomyini | *Otomys angoniensis* | TM123 | Female | Necsa - 2016 | 110 | 100 | 1001 | 0.0255 |  |
| Otomyini | *Otomys angoniensis* | TM22700 | Male | Necsa - 2016 | 110 | 100 | 1001 | 0.023 |  |
| Otomyini | *Otomys angoniensis* | TM22876 | Male | Necsa - 2016 | 110 | 100 | 1001 | 0.021 |  |
| Otomyini | *Otomys angoniensis* | TM24415 | Male | Necsa - 2016 | 110 | 100 | 1001 | 0.0194 |  |
| Otomyini | *Otomys angoniensis* | TM25623 | Male | Necsa - 2016 | 110 | 100 | 1001 | 0.022 |  |
| Otomyini | *Otomys angoniensis* | TM35027 | Male | Necsa - 2016 | 110 | 100 | 1001 | 0.0212 |  |
| Otomyini | *Otomys angoniensis* | TM44987 | Female | Necsa - 2016 | 110 | 100 | 1001 | 0.0195 |  |
| Otomyini | *Otomys angoniensis* | TM4504 | Male | Necsa - 2016 | 110 | 100 | 1001 | 0.0246 |  |
| Otomyini | *Otomys angoniensis* | TM45584 | Female | Necsa - 2016 | 110 | 100 | 1001 | 0.0207 |  |
| Otomyini | *Otomys angoniensis* | TM6485 | Female | Necsa - 2016 | 110 | 100 | 1001 | 0.024 |  |
| Otomyini | *Otomys auratus* | TM10282 | Female | Necsa - 2015 | 100 | 100 | 1001 | 0.0214 |  |
| Otomyini | *Otomys auratus* | TM15012 | Female | Necsa - 2015 | 100 | 100 | 1001 | 0.0202 |  |
| Otomyini | *Otomys auratus* | TM19287 | Male | Necsa - 2015 | 100 | 100 | 1001 | 0.0239 |  |
| Otomyini | *Otomys auratus* | TM2916 | Female | Necsa - 2015 | 100 | 100 | 1001 | 0.0226 |  |
| Otomyini | *Otomys auratus* | TM35182 | Male | Necsa - 2015 | 100 | 100 | 1001 | 0.0231 |  |
| Otomyini | *Otomys auratus* | TM3611 | Male | Necsa - 2016 | 110 | 100 | 1001 | 0.0225 |  |
| Otomyini | *Otomys auratus* | TM3614 | Female | Necsa - 2015 | 100 | 100 | 1001 | 0.0224 |  |
| Otomyini | *Otomys auratus* | TM41608 | Male | Necsa - 2015 | 100 | 100 | 1001 | 0.0213 |  |
| Otomyini | *Otomys auratus* | TM46864 | Female | Necsa - 2015 | 100 | 100 | 1001 | 0.0212 |  |
| Otomyini | *Otomys auratus* | TM7597 | Male | Necsa - 2015 | 100 | 100 | 1001 | 0.0214 |  |
| Otomyini | *Otomys auratus* | TM9052 | Female | Necsa - 2015 | 100 | 100 | 1001 | 0.0215 |  |
| Otomyini | *Otomys barbouri* | PM1933-2758 | Female | CIRIMAT-2016 | 130 | 180 | 2031 | 0.019608 |  |
| Otomyini | *Otomys barbouri* | PM1933-2759 | Female | CIRIMAT-2016 | 130 | 180 | 1789 | 0.019608 |  |
| Otomyini | *Otomys barbouri* | PM1933-2760 | Male | CIRIMAT-2017 | 130 | 180 | 2296 | 0.019608 |  |
| Otomyini | *Otomys barbouri* | PM1933-2762 | Male | CIRIMAT-2016 | 130 | 180 | 2122 | 0.019608 |  |
| Otomyini | *Otomys barbouri* | PM1933-2764 | Male | CIRIMAT-2016 | 130 | 180 | 2003 | 0.019608 |  |
| Otomyini | *Otomys barbouri* | PM1933-2765 | Male | CIRIMAT-2017 | 130 | 180 | 1971 | 0.019608 |  |
| Otomyini | *Otomys barbouri* | PM1933-2766 | Male | CIRIMAT-2017 | 130 | 180 | 1824 | 0.019608 |  |
| Otomyini | *Otomys helleri* | PM1972-218 | Male | CIRIMAT-2017 | 130 | 180 | 2298 | 0.019608 |  |
| Otomyini | *Otomys helleri* | PM1972-220 | Male | CIRIMAT-2016 | 130 | 180 | 2124 | 0.019608 |  |
| Otomyini | *Otomys helleri* | PM1972-221 | Male | CIRIMAT-2017 | 130 | 180 | 2116 | 0.019608 |  |
| Otomyini | *Otomys helleri* | PM1972-223 | Female | CIRIMAT-2017 | 130 | 180 | 2044 | 0.019608 |  |
| Otomyini | *Otomys helleri* | PM1972-224 | Female | CIRIMAT-2016 | 130 | 180 | 2148 | 0.019608 |  |
| Otomyini | *Otomys helleri* | PM1972-225 | Female | CIRIMAT-2017 | 130 | 180 | 2144 | 0.019608 |  |
| Otomyini | *Otomys helleri* | PM1972-226 | Female | CIRIMAT-2016 | 130 | 180 | 2148 | 0.019608 |  |
| Otomyini | *Otomys helleri* | PM1972-227 | Female | CIRIMAT-2016 | 130 | 180 | 2028 | 0.019608 |  |
| Otomyini | *Otomys helleri* | PM1972-228 | Male | CIRIMAT-2016 | 130 | 180 | 2124 | 0.019608 |  |
| Otomyini | *Otomys sloggetti* | TM1203 | Male | Necsa - 2016 | 110 | 100 | 1001 | 0.0219 |  |
| Otomyini | *Otomys sloggetti* | TM12915 | Female | Necsa - 2016 | 110 | 100 | 1001 | 0.0216 |  |
| Otomyini | *Otomys sloggetti* | TM13730 | Female | Necsa - 2016 | 110 | 100 | 1001 | 0.0212 |  |
| Otomyini | *Otomys sloggetti* | TM16519 | Male | Necsa - 2016 | 110 | 100 | 1001 | 0.0222 |  |
| Otomyini | *Otomys sloggetti* | TM22664 | Male | Necsa - 2016 | 110 | 100 | 1001 | 0.023 |  |
| Otomyini | *Otomys sloggetti* | TM22670 | Female | Necsa - 2016 | 110 | 100 | 1001 | 0.021 |  |
| Otomyini | *Otomys sloggetti* | TM22676 | Female | Necsa - 2016 | 110 | 100 | 1001 | 0.023 |  |
| Otomyini | *Otomys sloggetti* | TM22684 | Male | Necsa - 2016 | 110 | 100 | 1001 | 0.0212 |  |
| Otomyini | *Otomys sloggetti* | TM7606 | Male | Necsa - 2016 | 110 | 100 | 1001 | 0.0212 |  |
| Otomyini | *Otomys sloggetti* | TM7780 | Male | Necsa - 2016 | 110 | 100 | 1001 | 0.0226 |  |
| Otomyini | *Otomys sloggetti* | TM7781 | Female | Necsa - 2016 | 110 | 100 | 1001 | 0.0209 |  |
| Neotominae | *Peromyscus leucopus* | AN002 | Male | Necsa - 2019 | 100 | 100 | 1001 | 0.015585 |  |
| Neotominae | *Peromyscus leucopus* | AN003 | Male | Necsa - 2019 | 100 | 100 | 1001 | 0.015585 |  |
| Neotominae | *Peromyscus leucopus* | AN004 | Male | Necsa - 2019 | 100 | 100 | 1001 | 0.015585 |  |
| Neotominae | *Peromyscus leucopus* | AN005 | Male | Necsa - 2019 | 100 | 100 | 1001 | 0.01665 |  |
| Neotominae | *Peromyscus leucopus* | AN006 | Male | Necsa - 2019 | 100 | 100 | 1001 | 0.015585 |  |
| Neotominae | *Peromyscus leucopus* | LN17B | Male | Necsa - 2018 | 100 | 100 | 1001 | 0.016360 |  |
| Neotominae | *Peromyscus leucopus* | LN17D | Male | Necsa - 2018 | 100 | 100 | 1001 | 0.016360 |  |
| Neotominae | *Peromyscus leucopus* | LN17G | Male | Necsa - 2018 | 100 | 100 | 1001 | 0.016360 |  |
| Neotominae | *Peromyscus leucopus* | LN17H | Male | Necsa - 2018 | 100 | 100 | 1001 | 0.016749 |  |
| Neotominae | *Peromyscus leucopus* | LN17J | Female | Necsa - 2018 | 100 | 100 | 1001 | 0.016360 |  |
| Neotominae | *Peromyscus leucopus* | LN17N | Female | Necsa - 2018 | 100 | 100 | 1001 | 0.016360 |  |
| Neotominae | *Peromyscus leucopus* | LNF1104ak | Female | Necsa - 2019 | 100 | 100 | 1001 | 0.016263 |  |
| Neotominae | *Peromyscus leucopus* | LNF1112ah | Male | Necsa - 2019 | 100 | 100 | 1001 | 0.015382 |  |
| Neotominae | *Peromyscus leucopus* | LNF1112aj | Male | Necsa - 2019 | 100 | 100 | 1001 | 0.014287 |  |
| Neotominae | *Peromyscus leucopus* | LNF112ai | Male | Necsa - 2019 | 100 | 100 | 1001 | 0.015368 |  |
| Neotominae | *Peromyscus leucopus* | LNF1153a3 | Female | Necsa - 2019 | 100 | 100 | 1001 | 0.016036 |  |
| Neotominae | *Peromyscus leucopus* | LNF1155bq | Male | Necsa - 2019 | 100 | 100 | 1001 | 0.017183 |  |
| Neotominae | *Peromyscus leucopus* | LNF1159ag | Male | Necsa - 2019 | 100 | 100 | 1001 | 0.015043 |  |
| Neotominae | *Peromyscus leucopus* | LNF1159au | Male | Necsa - 2019 | 100 | 100 | 1001 | 0.015165 |  |
| Neotominae | *Peromyscus leucopus* | LNF1159bn | Male | Necsa - 2019 | 100 | 100 | 1001 | 0.015165 |  |
| Neotominae | *Peromyscus leucopus* | LNF1160al | Male | Necsa - 2019 | 100 | 100 | 1001 | 0.015382 |  |
| Neotominae | *Peromyscus leucopus* | LNF1160am | Male | Necsa - 2019 | 100 | 100 | 1001 | 0.016036 |  |
| Neotominae | *Peromyscus leucopus* | LNF1160aq | Male | Necsa - 2019 | 100 | 100 | 1001 | 0.015165 |  |
| Neotominae | *Peromyscus leucopus* | LNF124 | - | Necsa - 2019 | 100 | 100 | 1001 | 0.016738 |  |
| Neotominae | *Peromyscus leucopus* | LNF135 | - | Necsa - 2019 | 100 | 100 | 1001 | 0.016036 |  |
| Neotominae | *Peromyscus leucopus* | LNF145 | - | Necsa - 2019 | 100 | 100 | 1001 | 0.015165 |  |
| Neotominae | *Peromyscus maniculatus* | AN001 | Male | Necsa - 2019 | 100 | 100 | 1001 | 0.015585 |  |
| Neotominae | *Peromyscus maniculatus* | AN007 | - | Necsa - 2019 | 100 | 100 | 1001 | 0.015585 |  |
| Neotominae | *Peromyscus maniculatus* | AN008 | Female | Necsa - 2019 | 100 | 100 | 1001 | 0.015200 |  |
| Neotominae | *Peromyscus maniculatus* | AN009 | Female | Necsa - 2019 | 100 | 100 | 1001 | 0.014490 |  |
| Neotominae | *Peromyscus maniculatus* | LN17A | Male | Necsa - 2018 | 100 | 100 | 1001 | 0.016360 |  |
| Neotominae | *Peromyscus maniculatus* | LN17E | Male | Necsa - 2018 | 100 | 100 | 1001 | 0.016365 |  |
| Neotominae | *Peromyscus maniculatus* | LN17I | Male | Necsa - 2018 | 100 | 100 | 1001 | 0.016360 |  |
| Neotominae | *Peromyscus maniculatus* | LN17L | Male | Necsa - 2018 | 100 | 100 | 1001 | 0.016360 |  |
| Neotominae | *Peromyscus maniculatus* | ME0817_001 | Male | Necsa - 2018 | 100 | 100 | 1001 | 0.016360 |  |
| Neotominae | *Peromyscus maniculatus* | ME0817_002 | Female | Necsa - 2018 | 100 | 100 | 1001 | 0.016360 |  |
| Neotominae | *Peromyscus maniculatus* | ME0817_004 | Male | Necsa - 2018 | 100 | 100 | 1001 | 0.016360 |  |
| Neotominae | *Peromyscus maniculatus* | ME0817_005 | Male | Necsa - 2018 | 100 | 100 | 1001 | 0.016360 |  |
| Neotominae | *Peromyscus maniculatus* | ME0817_011 | Male | Necsa - 2018 | 100 | 100 | 1001 | 0.016360 |  |
| Neotominae | *Peromyscus maniculatus* | ME0817_018 | Female | Necsa - 2018 | 100 | 100 | 1001 | 0.016360 |  |
| Neotominae | *Peromyscus maniculatus* | ME0817_022 | Female | Necsa - 2018 | 100 | 100 | 1001 | 0.016360 |  |
| Neotominae | *Peromyscus maniculatus* | ME0817_024 | Female | Necsa - 2018 | 100 | 100 | 1001 | 0.016360 |  |
| Neotominae | *Peromyscus maniculatus* | ME0817_026 | Male | Necsa - 2018 | 100 | 100 | 1001 | 0.016360 |  |
| Neotominae | *Peromyscus maniculatus* | ME0817_046 | Male | Necsa - 2018 | 100 | 100 | 1001 | 0.016360 |  |
| Neotominae | *Peromyscus maniculatus* | ME0817_050 | Female | Necsa - 2018 | 100 | 100 | 1001 | 0.016360 |  |
| Neotominae | *Peromyscus maniculatus* | MEF1134at | Female | Necsa - 2019 | 100 | 100 | 1001 | 0.015089 |  |
| Neotominae | *Peromyscus maniculatus* | MEF1146av | Female | Necsa - 2019 | 100 | 100 | 1001 | 0.015200 |  |
| Neotominae | *Peromyscus maniculatus* | MEF1146aw | Female | Necsa - 2019 | 100 | 100 | 1001 | 0.015200 |  |
| Neotominae | *Peromyscus maniculatus* | MEF1146ax | Female | Necsa - 2019 | 100 | 100 | 1001 | 0.015200 |  |
| Neotominae | *Peromyscus maniculatus* | MEF1148d | Male | Necsa - 2019 | 100 | 100 | 1001 | 0.014808 |  |
| Neotominae | *Peromyscus maniculatus* | MEF1149aL | Male | Necsa - 2019 | 100 | 100 | 1001 | 0.014808 |  |
| Neotominae | *Peromyscus maniculatus* | MEF1149am | Male | Necsa - 2019 | 100 | 100 | 1001 | 0.015200 |  |
| Neotominae | *Peromyscus maniculatus* | MEF1149L | Female | Necsa - 2019 | 100 | 100 | 1001 | 0.015089 |  |
| Praomyini | *Praomys hartwigi* | MNHN CG 2011_218 | Female | Necsa - 2018 | 100 | 100 | 1001 | 0.018414 |  |
| Praomyini | Praomys hartwigi | MNHN CG 2011_221 | Male | Necsa - 2018 | 100 | 100 | 1001 | 0.019003 |  |
| Praomyini | Praomys hartwigi | MNHN CG 2011_236 | Male | Necsa - 2018 | 100 | 100 | 1001 | 0.019003 |  |
| Praomyini | *Stenocephalemys albipes* | MNHN 1972_167 | Male | Necsa - 2018 | 100 | 100 | 1001 | 0.02096 |  |
| Praomyini | *Stenocephalemys albipes* | MNHN 1972_186 | Female | Necsa - 2018 | 100 | 100 | 1001 | 0.020209 |  |
| Praomyini | *Stenocephalemys albipes* | MNHN 1972_187 | Female | Necsa - 2018 | 100 | 100 | 1001 | 0.020307 |  |
| Praomyini | *Stenocephalemys albocaudata* | MNHN 1972_208 | Male | Necsa - 2018 | 100 | 100 | 1001 | 0.022234 |  |
| Praomyini | *Stenocephalemys albocaudata* | MNHN 1972_217 | Female | Necsa - 2018 | 100 | 100 | 1001 | 0.023557 |  |
| Praomyini | *Stenocephalemys griseicauda* | MNHN 1972_155 | Male | Necsa - 2018 | 100 | 100 | 1001 | 0.022732 |  |
| Praomyini | *Stenocephalemys griseicauda* | MNHN 1972_158 | Female | Necsa - 2018 | 100 | 100 | 1001 | 0.019074 |  |
| Praomyini | *Stenocephalemys griseicauda* | MNHN 1972_162 | Female | Necsa - 2018 | 100 | 100 | 1001 | 0.020987 |  |

**Supplementary file S6:** R-script (code) for running the Phylogenetic Generalized Least Squares (PGLS). Comments and information about the scrip (not run) are preceded by a hash symbol (#). GLS and ECV_cm3 are depicted by **6**:**7** on the script.

### Loading the packages into R environment

library(geiger)

library(nlme)

library(phytools)

library(tidyverse)

library(car)

library (ggplot2)

library(ggpubr)

library(emmeans)

library(rstatix)

### Filling in the phylogenetic tree of the study species

NJ_Oto="(((((angoniensis:0.03958621,auratus:0.03623826)0.2930:0.00239071,sloggetti:0.04376772)0.2560:0.00189915,helleri:0.03701508)0.4760:0.00750619,barbouri:0.05691242)0.9940:0.03636444,(maniculatus:0.04468228,leucopus:0.05600263)0.9990:0.04258696,(hartwigi:0.05527921,(albocaudata:0.02529677,(griseicauda:0.01844171,albipes:0.03872090)0.7620:0.00786460)0.9880:0.02947775)0.9980:0.03908674);"

NJ_Oto.tree <-read.tree(text=NJ_Oto)

### Specifying path to the file containing raw data for the study

setwd ("C:/Users/taylorpj/Dropbox/Papers/Alu_endocranium/Combined MS")

endocranium<-read.csv("Endocranium_vol_September2019Updated.csv")

attach(endocranium)

### Aggegate dataset into means

endocranium_final<-aggregate(endocranium [, **6**:**7**], list(endocranium$Species), mean)

View(endocranium_final)

colnames(endocranium_final)[**1**]<-"Species"

endocranium_final

attach(endocranium_final)

### Phylogenetic regression of ECV and skull length

pglsModel <- gls(ECV_cm3 ~ GLS, correlation =corBrownian(phy = NJ_Oto.tree, form = ~Species),

data = endocranium_final, method = "ML")

summary(pglsModel)

### Add variable for Subfamily

subfamily<-c(**1**,**1**,**2**,**2**,**2**,**1**,**1**,**2**,**3**,**3**,**2**)

endocranium_final$subfamily<-subfamily

View(endocranium_final)

par(mar = c(**4.1**, **4.4**, **4.1**, **1.9**))

###Plots

plot(ECV_cm3~ GLS,pch=c(**16**,**16**,**17**,**17**,**17**,**16**,**16**,**17**,**15**,**15**,**17**),col=factor (subfamily),

xlab= "Skull length (GLS)", ylab = "Endocranial volume (cm3)")

abline(lm(ECV_cm3 ~ GLS))

text(GLS, ECV_cm3, Species, font = **1.5**, cex = **0.9**)

### Subset Otomys

Otomys <- subset(endocranium, Genus == "Otomys")

attach(Otomys)

## Conduct analysis of covariance for ECV and elevation for Otomys

ancova1 <- aov(log(ECV_cm3) ~ as.factor(Elevation) + log(GLS))

shapiro.test(resid(aov(log(ECV_cm3) ~ as.factor(Elevation) + log(GLS), Otomys)))

Anova(ancova1, type = "III")

ggscatter(Otomys, x = "GLS", y = "ECV_cm3",color = "Elevation", add = "reg.line")+ stat_regline_equation(aes(label = paste(..eq.label.., ..rr.label.., sep = "~~~~"), color = Elevation))

## log variables for plots

Otomys$GLS_log <- log(Otomys$GLS)

Otomys$ECV_cm3_log <- log (Otomys$ECV_cm3)

ggscatter(Otomys, x = "GLS_log", y = "ECV_cm3_log",color = "Elevation", add = "reg.line")+ stat_regline_equation(aes(label = paste(..eq.label.., ..rr.label.., sep = "~~~~"), color = Elevation))

## Conduct analysis of covariance for ECV and elevation for high/low Peromyscus

ancova2 <- aov(log(ECV_cm3) ~ as.factor(Elevation) + log(GLS))

shapiro.test(resid(aov(log(ECV_cm3) ~ as.factor(Elevation) + log(GLS), Pero)))

Anova(ancova2, type = "III")

ggscatter(Pero, x = "GLS", y = "ECV_cm3",color = "Elevation", add = "reg.line")+ stat_regline_equation(aes(label = paste(..eq.label.., ..rr.label.., sep = "~~~~"), color = Elevation))

## log variables for plots

Pero$GLS_log <- log(Pero$GLS)

Pero$ECV_cm3_log <- log (Pero$ECV_cm3)

ggscatter(Pero, x = "GLS_log", y = "ECV_cm3_log",color = "Elevation", add = "reg.line")+ stat_regline_equation(aes(label = paste(..eq.label.., ..rr.label.., sep = "~~~~"), color = Elevation))

## Conduct analysis of covariance for ECV and elevation for high/low/lab/wild Peromyscus

ancova3 <- aov(log(ECV_cm3) ~ as.factor(description) + log(GLS))

Anova(ancova3, type = "III")

ggscatter(Pero, x = "GLS_log", y = "ECV_cm3_log",color = "description", add = "reg.line")+ stat_regline_equation(aes(label = paste(..eq.label.., ..rr.label.., sep = "~~~~"), color = description))

## posthoc test of adjusted ECV_cm3 means

library(dplyr)

# group_by() on department

grp_Pero <- Pero %>% group_by(description)

grp_Pero

emmeans_test(data = Pero, formula = ECV_cm3 ~ description, covariate = GLS, p.adjust.method = "fdr")

### Subset Praomys/Stenocephalemys

Praomyini <- subset(endocranium, Genus %in% c('Praomys','Stenocephalemys'))

attach(Praomyini)

## Conduct analysis of covariance for ECV and elevation for high/low Praomyini

ancova5 <- aov(log(ECV_cm3) ~ as.factor(Elevation) + log(GLS))

shapiro.test(resid(aov(log(ECV_cm3) ~ as.factor(Elevation) + log(GLS), Praomyini)))

Anova(ancova5, type = "III")

ggscatter(Praomyini, x = "GLS", y = "ECV_cm3",color = "Elevation", add = "reg.line")+ stat_regline_equation(aes(label = paste(..eq.label.., ..rr.label.., sep = "~~~~"), color = Elevation))

## log variables for plots

Praomyini$GLS_log <- log(Praomyini$GLS)

Praomyini$ECV_cm3_log <- log (Praomyini$ECV_cm3)

ggscatter(Praomyini, x = "GLS_log", y = "ECV_cm3_log",color = "Elevation", add = "reg.line")+ stat_regline_equation(aes(label = paste(..eq.label.., ..rr.label.., sep = "~~~~"), color = Elevation))
